# The *Fraxinus mandshurica* genome reveals a dual adaptive strategy linking rapidly evolving immune receptors and structural fortification

**DOI:** 10.48130/forres-0026-0026

**Published:** 2026-08-31

**Authors:** Jialin Yan, Shuai Yang, Qiang Feng, Liming He, Ying Xin, Fenghui Qi, Yaguang Zhan, Fansuo Zeng

**Affiliations:** 1 State Key Laboratory of Tree Genetics and Breeding, Northeast Forestry University, Harbin 150040, China; 2 College of Life Science, Northeast Forestry University, Harbin 150040, China; 3 College of Forestry, Northeast Forestry University, Harbin 150040, China

**Keywords:** *Fraxinus mandshurica*, Genome assembly, NLR genes, Transcriptional regulation, Comparative genomics, Lignification

## Abstract

*Fraxinus mandshurica* exhibits high tolerance to ash dieback and superior wood mechanical properties, yet the genomic basis of these dual traits remains incompletely understood. Here, we present a chromosome-scale genome assembly of the elite clone 'M8' and integrate comparative genomics with temporal transcriptomics to investigate its adaptive architecture. We show that recent tandem and proximal duplications have contributed to the diversification and candidate positive selection of the NLR immune repertoire. Transcriptomic profiling reveals a stage-associated deployment pattern, in which expressed NLR genes are enriched during the Transition Stage, consistent with reinforced immune surveillance during rapid xylem expansion. As development progresses, the Latewood Stage shifts toward phenylpropanoid metabolism, lignification-related programs, and structural reinforcement. We identify and experimentally validate a latewood-recruited *FmNAC104–FmPRX1* candidate regulatory module, in which FmNAC104 binds to and activates the *FmPRX1* promoter and promotes endogenous *FmPRX1* expression. Formal NAC/PRX gene family analysis further supports an enlarged NAC/PRX repertoire relative to *P. trichocarpa* and clarifies the orthogroup status of the *FmNAC104–FmPRX1* module among related ash species. Collectively, our findings support a 'fortified defense' working model in which *F. mandshurica* temporally coordinates immune-receptor diversification with latewood-associated structural reinforcement. This study provides a genomic resource and candidate targets for breeding programs aimed at improving wood quality and disease resilience in *Fraxinus*.

## Introduction

*Fraxinus mandshurica* (Manchurian ash), a diploid (2*n* = 46) deciduous tree in the Oleaceae family, is an ecologically and economically important species in Northeast Asian temperate forests and is celebrated for two distinct yet equally vital adaptive traits: its superior wood quality and its remarkable disease resilience^[1,2]^. Economically, its timber is highly prized for exceptional strength, durability, and shock resistance, making it a critical resource for industries facing growing timber scarcity. Ecologically, its robust adaptability to cold, high-altitude environments and its resilience to biotic stresses position it as an important resource for both conservation and forestry in challenging climates^[3,4]^.

A defining feature of *F. mandshurica* is its ring-porous wood anatomy, characterized by a stark seasonal differentiation between large, conductive earlywood vessels and dense, mechanically supportive latewood fibers^[5]^. This structure reflects an evolutionary trade-off, balancing rapid spring growth with the structural integrity required for a long-lived perennial. The high density and lignin content of its latewood are fundamental to its superior material properties. Concurrently, and in stark contrast to many of its congeners, *F. mandshurica* exhibits profound resistance to devastating pathogens, most notably *Hymenoscyphus fraxineus*, the causal agent of ash dieback that has decimated European ash (*Fraxinus excelsior*) populations^[6−8]^. This dual excellence in both structural performance and pathogen defense suggests an underlying integrated genetic architecture that has been finely tuned by evolution^[9]^.

To defend against biotic threats, plants rely on a sophisticated multi-layered immune system. Central to this defense is a surveillance network of intracellular receptors, particularly Nucleotide-binding Leucine-rich repeat (NLR) proteins, which recognize pathogen effectors and trigger robust immune responses^[10]^. The size, diversity, and evolutionary dynamics of the NLR repertoire are key determinants of a plant's resistance spectrum^[11]^. However, while the expansion of these immune receptors is often associated with adaptation to fluctuating pathogen pressures, how this 'active' immune system has evolved in concert with preformed structural defenses in long-lived trees remains a frontier in understanding forest resilience^[12]^.

Complementing this active surveillance, the plant secondary cell wall serves as a critical structural nexus connecting disease resilience with wood quality. As the primary component of wood, its composition—particularly the deposition of lignin—directly determines timber's physical properties, such as density and strength^[13]^. Simultaneously, this lignified wall constitutes a major preformed physical barrier against pathogen invasion, a constitutive defense mechanism^[9]^. Furthermore, lignification can be induced at infection sites as part of the immune response. Therefore, the gene networks governing secondary cell wall biosynthesis, particularly those active during latewood formation, represent potential evolutionary targets where selection pressures for mechanical support and defensive fortification converge^[14,15]^.

Despite the ecological and economic importance of *Fraxinus*, genomic research has been constrained. While preliminary genome assemblies exist, they have generally not focused on elite breeding clones with well-characterized wood-quality and disease-resilience traits, limiting their direct utility for dissecting the genomic basis of superior germplasm performance^[16,17]^. 'M8' is an elite *F. mandshurica* clone selected for its outstanding trunk straightness, superior wood density, and broad-spectrum disease resistance. As a premier maternal parent extensively used in interspecific hybridization programs, 'M8' represents a critical germplasm resource for ash breeding. A high-quality, chromosome-level reference genome of this elite clone is thus essential, not only to overcome the limitations of fragmentation in previous assemblies but to specifically decode the genetic underpinnings of the 'dual adaptations' observed in superior breeding lines. Such a resource would enable the precise identification of regulatory networks for wood formation and the evolutionary history of the immune gene arsenal in the context of elite germplasm^[18]^.

Here, we present a chromosome-level reference genome of the elite *F. mandshurica* clone 'M8' and integrate comparative genomics with high-resolution xylem transcriptomics to decode the genetic basis of its dual resilience. We examine how immune-receptor diversification and latewood-associated regulatory programs may jointly contribute to disease resilience and wood quality. We show that recent tandem and proximal duplications have fueled the rapid diversification of the NLR immune repertoire, establishing a heightened surveillance network within the vascular system. Concurrently, we delineate the transcriptional architecture of wood formation, identifying a latewood-recruited *FmNAC104–FmPRX1* candidate regulatory module, for which promoter binding, transcriptional activation, and native upregulation were experimentally validated. Synthesizing these findings, we propose a model of 'fortified defense' where the species integrates an expanded immune arsenal with constitutive structural reinforcement. This study not only provides genomic and regulatory insights into the adaptive basis of *F. mandshurica*'s exceptional adaptability but also provides a critical genomic blueprint for breeding programs aimed at improving wood quality and disease resilience in *Fraxinus*.

## Materials and methods

### Plant materials and DNA sequencing

Fresh, mature leaves were collected from a single individual of the elite female clonal line 'M8' of *F. mandshurica* at the Mao'ershan Experimental Forest (Heilongjiang, China). High-molecular-weight genomic DNA was extracted using a modified CTAB method^[19]^, incorporating RNase A treatment and extended chloroform : isoamyl alcohol purification to remove contaminants^[20]^. DNA quality and concentration were verified using a NanoDrop 2000 spectrophotometer (Thermo Fisher Scientific), a Qubit fluorometer (Invitrogen), and 0.8% agarose gel electrophoresis. SMRTbell libraries were constructed using the SMRTbell Express Template Prep Kit 2.0 (Pacific Biosciences) and sequenced on the PacBio Sequel II platform to generate high-fidelity long reads for *de novo* assembly.

To facilitate chromosome-level scaffolding, Hi-C libraries were prepared from fresh leaf tissues of the same clonal line 'M8'. Samples were cross-linked with 1% formaldehyde via vacuum infiltration and digested with the restriction enzyme *Mbo*I^[21,22]^. DNA fragments were biotinylated using biotin-14-dATP, proximity-ligated, and purified to reverse cross-links. Biotinylated fragments were enriched using streptavidin-coated magnetic beads^[23]^ and sequenced on the Illumina HiSeq X Ten platform (150-bp paired-end) to capture genome-wide chromatin interactions^[24]^. In addition, an Illumina whole-genome sequencing library was constructed from the same clonal line and sequenced using paired-end reads. These short reads were used for k-mer-based genome characterization, read-based assembly validation, and Merqury consensus-quality assessment.

### Transcriptome sequencing

#### Iso-Seq and RNA-seq for genome annotation

To generate a comprehensive transcript reference, total RNA was extracted from a pooled sample of diverse tissues (leaves, phloem, and xylem) of the 'M8' clone using the RNeasy Plant Mini Kit (Qiagen). RNA integrity (RIN ≥ 8) was confirmed using an Agilent 2100 Bioanalyzer. For full-length transcript sequencing, cDNA was synthesized using the SMARTer PCR cDNA Synthesis Kit (Takara Bio) and size-selected with the BluePippin system (Sage Science). SMRTbell libraries were constructed and sequenced on the PacBio Sequel II platform^[25]^. Additionally, to support error correction and quantification, a poly(A)-enriched short-read library was constructed from the same RNA pool using the NEBNext Ultra II RNA Library Prep Kit (New England Biolabs) and sequenced on the Illumina HiSeq X Ten platform (150-bp paired-end).

#### Time-series RNA-seq of developing xylem

To investigate transcriptional dynamics during wood formation, developing xylem tissues were harvested from the stems of three-year-old 'M8' clonal seedlings at the Mao'ershan Experimental Forest. Samples were collected at weekly intervals from April 19 to September 5, 2023, covering the seasonal transition from earlywood to latewood formation. A total of 18 time points were sampled, with three biological replicates planned for each time point. After RNA quality control, library quality assessment, and sequencing-read filtering, 50 high-quality samples were retained for downstream time-series transcriptomic analysis. Libraries were constructed using the NEBNext Ultra II RNA Library Prep Kit and sequenced on the DNBSEQ-T7 platform (100 bp paired-end)^[26]^.

### Transcriptome data processing and network construction

#### Data processing and gene quantification

PacBio raw data were processed using the Iso-Seq 3 pipeline in SMRT Link (v10.1) to generate high-quality full-length non-chimeric (FLNC) reads^[27]^. Short reads were quality-filtered using fastp (v0.23.2)^[28]^. For genome annotation, clean short reads were mapped to the *F. mandshurica* genome using HISAT2 (v2.2.1)^[29]^ and assembled with StringTie (v2.2.1)^[30]^. These assemblies were merged with FLNC reads to generate the final gene models. For the time-series analysis, clean reads from the DNBSEQ-T7 platform were aligned to the reference genome using HISAT2. Gene-level read counts were quantified using featureCounts (v2.0.1)^[31]^. Transcript abundance was also summarized as TPM for expression visualization and clustering analyses.

#### Differential expression and network inference

The time-ordered expression matrix was normalized, and differentially expressed genes (DEGs) were identified using the DESeq2 package (v1.38.3) in R^[32]^ with thresholds of FDR < 0.05 and |log_2_ fold change| > 1, unless otherwise specified. Time-ordered gene co-expression networks (TO-GCNs) were constructed following the methodology described previously^[33]^. Furthermore, putative transcription factor–target gene regulatory relationships were inferred using the GENIE3 algorithm^[34]^, which utilizes a random forest-based approach to predict regulatory links from expression data.

### Genome assembly, annotation, and assembly quality assessment

#### Genome assembly

The assembly process commenced with rigorous quality control of raw PacBio data to generate high-accuracy circular consensus sequencing (CCS) reads. Contig assembly was performed using hifiasm (v0.18.1), a long-read assembler optimized for HiFi reads, to construct preliminary contigs^[35]^. The assembled contigs were then anchored to chromosomes using Hi-C data through the Juicer (v1.6) pipeline, followed by refinement with the 3D-DNA method. This approach enabled efficient clustering, ordering, and orienting of contig sequences into chromosome-length scaffolds^[36,37]^. After Hi-C assembly, manual curation was performed to finalize the assembly into pseudochromosomes. The final chromosome-scale assembly was further inspected using Hi-C contact maps to evaluate chromosome-level ordering, orientation, and potential misjoins.

#### Assembly quality assessment

K-mer-based genome characterization was performed using filtered Illumina whole-genome sequencing reads to estimate genome size, sequencing depth, heterozygosity, and repeat content. GenomeScope2 (v2.0.1) was used to model the k-mer frequency distribution (Supplementary Fig. S1)^[38]^. Assembly consensus accuracy was assessed using Merqury (v1.3), which estimates consensus quality value (QV), error rate, and k-mer completeness based on read-derived k-mers^[39]^. Chromosome-level QV values were calculated for the 23 pseudochromosomes and summarized in Fig. 1c and Supplementary Table S1. BUSCO (v5.5.0) was used to evaluate genome and protein-set completeness against the Embryophyta odb10 dataset.

**Figure 1:**
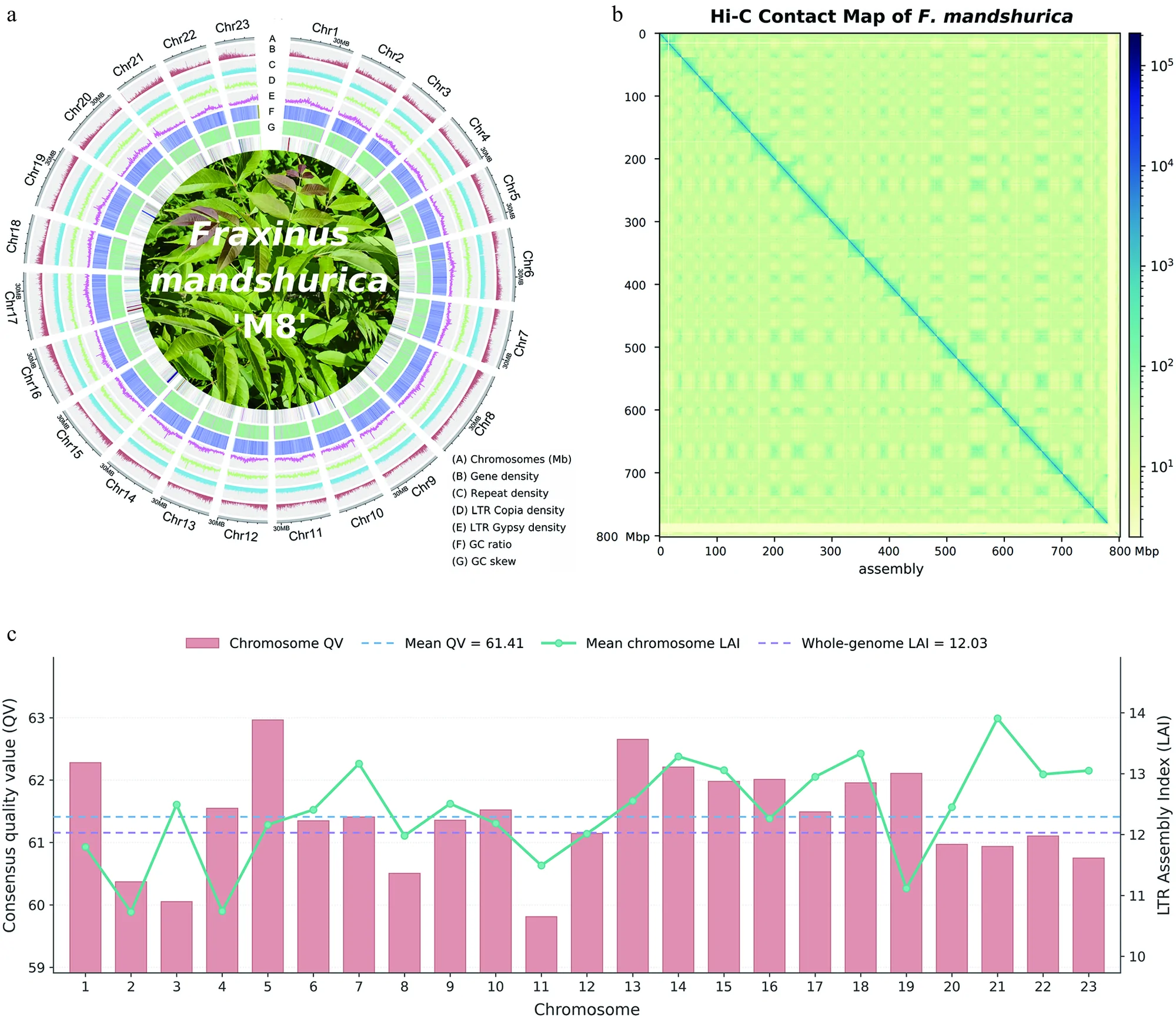
Assembly and landscape of the *F. mandshurica* genome. (a) Circos representation of genomic features across the 23 pseudochromosomes. Tracks are arranged from the periphery inward: (A) Karyotype of the assembled chromosomes (Mb). (B) Gene density. (C) Density of total transposable elements (TEs). (D) Distribution of Copia-like LTR retrotransposons. (E) Distribution of Gypsy-like LTR retrotransposons. (F) GC content. (G) GC skew. All features were calculated in non-overlapping windows of 500 kb. The innermost lines depict intragenomic syntenic blocks. (b) Genome-wide Hi-C interaction map. The heatmap displays the interaction intensity of chromatin contacts, with the clear diagonal squares resolving the 23 pseudochromosomes, indicating a high-quality chromosome-scale assembly. (c) Chromosome-level assembly quality assessment showing Merqury consensus QV values and chromosome-averaged LTR Assembly Index (LAI). Dashed lines indicate the mean chromosome-level QV and the whole-genome LAI, respectively.

#### Repeat annotation and LAI assessment

Repetitive elements in the *F. mandshurica* genome were annotated through a multi-step process. First, repeat families were modeled *de novo* using RepeatModeler (v2.0.5)^[40,41]^, which generated a species-specific repeat library. This library was then combined with known repeat databases, including Repbase (v23.12)^[42]^ and Dfam (v3.6)^[43]^, and redundancies were removed to enhance accuracy. The annotation of repetitive elements was conducted using RepeatMasker (v4.1.6)^[40]^ and LTR_Retriever (v3.0.5)^[44]^. To evaluate the continuity of LTR-rich repetitive regions, the LTR Assembly Index (LAI) was calculated using LTR_Retriever based on intact and total LTR retrotransposon annotations^[45]^. Whole-genome and chromosome-level LAI values were summarized to assess repeat-region assembly quality.

#### Putative centromere-like and pericentromeric region analysis

To reassess the genomic features of putative centromere-like regions, each chromosome was divided into 50-kb non-overlapping windows. For each window, we calculated gene count per Mb, gene coverage, total TE fraction, LTR-Copia fraction, LTR-Gypsy fraction, other LTR fraction, SSR fraction, GC content, and gap ratio. Putative centromere-like windows were selected based on a composite window score integrating reduced gene density/gene coverage and enriched repeat- or microsatellite-related signals. These regions were visualized together with gap ratio and repeat tracks for all 23 chromosomes (Supplementary Fig. S2). Telomeric and candidate centromeric features were additionally inspected using quarTeT (v1.2.5)^[46]^, and candidate centromeric features were further validated with the same tool.

#### Gene prediction and functional annotation

Protein-coding genes were predicted on the soft-masked genome using the MAKER2 (v2.31.1)^[47]^ and PASA (v2.5.2)^[48]^ annotation pipelines. This approach integrated multiple lines of evidence: *ab initio* predictions were performed using Augustus (v3.3.3)^[49]^, SNAP (v2013)^[50]^, and GeneMark-ES (v4.68)^[51]^. Transcriptome-based predictions were incorporated using RNA-seq and Iso-Seq data, ensuring the accuracy of transcript structures. Homology-based predictions utilized high-quality protein datasets from Swiss-Prot to improve gene model reliability. The protein sequences obtained from structural annotation were compared against functional databases, including InterPro, Swiss-Prot, KEGG^[52]^, NR, TrEMBL, and COG, using BLASTP (v2.2.31)^[53]^ with an E-value threshold of 1e-05. Database-specific annotation rates were summarized in Supplementary Table S2.

#### Non-coding RNA (ncRNA) annotation

Different classes of ncRNAs were identified using specialized tools. Transfer RNAs (tRNAs) were predicted using tRNAscan-SE (v1.3.1)^[54]^. Ribosomal RNAs (rRNAs), small nuclear RNAs (snRNAs), and microRNAs (miRNAs) were identified by searching the genome against the Rfam database (v12.0)^[55]^ using Infernal (v1.1.2)^[56]^.

### Comparative genomic analysis

#### Genomic data sources

The genome assemblies and associated annotations for comparative genomic analyses were retrieved from publicly available databases. The dataset included species representing key evolutionary nodes: *S. oblata*^[57]^, *O. europaea*^[58]^, *F. pennsylvanica*^[16]^, *F. excelsior*^[59]^, *V. vinifera*^[60]^, *M. domestica*^[61]^, *Q. rubra*^[62]^, *J. mandshurica*^[63]^, *B. platyphylla*^[64]^, *A. thaliana*^[65]^, *P. trichocarpa*^[66]^, alongside the *F. mandshurica* assembly generated in this study (Supplementary Table S1).

#### Phylogenetic and evolutionary rate analysis

Orthologous and paralogous genes were identified using OrthoFinder (v2.4.0)^[67]^. Gene family expansions and contractions were detected using CAFE (v4.2.1)^[68]^. For phylogenetic analysis, nucleotide sequences were aligned with MAFFT (v7.450)^[69]^, trimmed using Gblocks (v0.91b)^[70]^, and the retained alignment blocks were concatenated for phylogenetic reconstruction. Phylogenetic trees were constructed using RAxML (v8)^[71]^ and visualized in FigTree (v1.4.4). Molecular clock and divergence times were estimated with PAML (v4.9i)^[72]^, utilizing the codeml and MCMCtree programs^[72]^. Divergence calibrations were derived from curated estimates in the TimeTree database^[73]^ and supplemented with three fossil-supported age constraints representing major nodes within Oleaceae and broader angiosperm lineages. These priors, with calibration ranges of 19.9–38.3, 38.1–51.2, and 75.8–96.6 Mya, were implemented as soft temporal constraints.

#### Whole-genome duplication and synteny analysis

Protein sequences were compared using BLASTP (v2.2.31) with an E-value threshold of 1e-05. Collinear blocks were identified using WGDI (v0.6.4)^[74]^, JCVI (v1.3.4)^[75]^, and MCScanX (v1.0)^[76]^. WGD events were inferred based on the distribution of synonymous substitution rates (K_s_) and 4DTv (transversion rate at four-fold degenerate sites) values of syntenic gene pairs.

#### Structural variation analysis

To characterize large-scale genomic rearrangements, the *F. mandshurica* genome was compared with other *Fraxinus* species. Homologous chromosomes were aligned using MUMmer (v4.0)^[77]^, and structural variations (SVs), including inversions, translocations, and copy number variations (CNVs), were identified using SyRI (v1.5)^[78]^. Gene expansions potentially driven by 'copy gain' events were specifically annotated to investigate lineage-specific adaptations.

### Gene duplication and positive selection analysis

Gene duplication events were categorized using DupGen_finder (v2.0)^[79]^, which classifies duplications into whole-genome (WGD), tandem (TD), proximal (PD), transposed (TRD), and dispersed (DD) types. This classification integrated collinearity information derived from MCScanX (v1.0) and pairwise protein alignments performed using BLASTP (v2.2.31; E-value ≤ 1e-05).

To assess the selection pressures acting on duplicated gene pairs, we calculated the rates of synonymous (*K_s_*) and nonsynonymous (*K_a_*) substitutions. For each pair, protein sequences were aligned, and the corresponding coding sequences were used to calculate *K_a_* and *K_s_* values with KaKs_Calculator (v3.0)^[80]^, employing the Yang-Nielsen (YN) model. The *K_a_*/*K_s_* ratio (*ω*) was used to infer the type of evolutionary selection acting on the duplicated pairs.

### Identification, domain validation, and phylogenetic analysis of NLR genes

NLR gene repertoires were identified from the whole-genome sequences of ten *Fraxinus* species: *Fraxinus americana*, *F. angustifolia*, *F. chinensis*, *F. excelsior*, *F. hupehensis*, *F. mandshurica*, *F. ornus*, *F. pennsylvanica*, *F. sogdiana*, and *F. velutina*. Genome data were sourced from^[17]^. Candidate NLR genes were predicted using NLR-Annotator (v2.1)^[81]^. This tool identifies NLRs by searching for conserved motifs and domains, including the NB-ARC domain (PF00931) and various LRR domains (PF00560, PF07723, PF13855)^[82]^, using Hidden Markov Model (HMM) profiles, often sourced from or comparable to those in the Pfam database^[83]^.

Predicted protein sequences were extracted and compared against a curated *Arabidopsis thaliana* NLR dataset^[84]^ via TAIR^[85]^. Reciprocal BLASTP searches (v2.2.31)^[53]^ were performed between candidate NLRs and the reference dataset with an E-value threshold of 1e-05. Domain architectures were further verified using NCBI CD-Search^[86]^. For each retained NLR candidate, the presence or absence of NB-ARC, LRR, TIR, CC, and RPW8-related domains was recorded, and candidates were classified into major NLR subtypes where possible. Sequences shorter than 50 amino acids or lacking canonical NB-ARC/LRR motifs were discarded.

Synonymous substitution rates (*K_s_*) among homologous NLRs were estimated with PAML (v4.9i). Multiple-sequence alignments were generated using ClustalX (v2.0)^[87]^, and a maximum likelihood (ML) phylogenetic tree was constructed using IQ-TREE2^[88]^ with automatic model selection (ModelFinder Plus, -m MFP). Tree topology was assessed using 1,000 ultrafast bootstrap replicates (-bb 1,000). The phylogenetic tree was visualized and annotated using iTOL (v6)^[89]^.

### Pathogen inoculation and disease quantification

Leaves were spot-inoculated with mycelial plugs (5 mm diameter) of *Fusarium iranicum* and *Colletotrichum sojae*, isolated from local environments. For each treatment, nine biological replicates were performed. Disease severity was quantified 10 d post-inoculation (dpi) by measuring the necrotic lesion area using Image-Pro Plus software (Media Cybernetics). Briefly, a spatial calibration was established within the software using a 20 cm reference scale included in each image to ensure accurate conversion of pixel counts to physical area (cm^2^). The boundaries of each necrotic lesion were manually outlined using the 'Area' measurement tool, and the absolute lesion area was automatically calculated based on the calibrated ratio. Statistical significance was assessed using Student's *t*-test, with *p* < 0.05 considered significant. Data are presented as mean ± SD.

### Molecular validation of the FmNAC104–FmPRX1 candidate regulatory module

#### NAC/PRX family identification and anchor mapping

To support the comparative interpretation of the *FmNAC104–FmPRX1* module, NAC and PRX family members were identified from the protein datasets of *F. mandshurica*, *F. excelsior*, *F. sogdiana*, *F. pennsylvanica*, and *P. trichocarpa*. After retaining one representative protein per gene and removing terminal stop codons, NAC and PRX candidates were identified using HMMER (v3.4)^[90]^ searches against the Pfam NAC/NAM domain profile (PF02365) and the peroxidase domain profile (PF00141), respectively. Candidate members were further verified using full Pfam scans and CDD/RPS-BLAST searches. The experimentally validated *FmNAC104* and *FmPRX1* sequences were mapped to their respective families and assigned to orthogroups using OrthoFinder (v2.4.0). All identified NAC/PRX genes, domain validation status, mapped anchors, and orthogroup members are listed in Supplementary Dataset S1.

#### Yeast one-hybrid (Y1H) assay

Direct binding of the *FmNAC104* protein (Chr15G001472.1) to the *FmPRX1* promoter (Chr01G001878.1) was tested using the GAL4-based yeast one-hybrid system. The full-length coding sequence of *FmNAC104* was cloned into the pGADT7-Rec2 vector, and the promoter fragment of *FmPRX1* was inserted into the pHIS2 vector using Seamless Assembly Cloning (Clone Smarter). The promoter of a non-target gene, *FmCAD1*, was similarly cloned into pHIS2 to serve as a negative control. The bait and prey constructs were co-transformed into *Saccharomyces cerevisiae* strain Y187. Transformants were initially selected on synthetic dropout medium lacking leucine and tryptophan (SD/-Leu/-Trp). Positive protein-DNA interactions were subsequently evaluated by spotting the co-transformants onto triple dropout medium lacking leucine, tryptophan, and histidine (SD/-Leu/-Trp/-His), supplemented with 60 mM of 3-amino-1,2,4-triazole (3-AT) to suppress background histidine leakage.

#### Transient dual-luciferase reporter assay

Transcriptional activation of the *FmPRX1* promoter by *FmNAC104* was quantified *in planta* using a dual-luciferase reporter assay^[91]^. The promoter fragment of *FmPRX1* was cloned into the pGreenII 0800-LUC reporter vector, while the full-length coding sequence of *FmNAC104* was inserted into the pGreenII 62-SK effector vector. Constructs were transformed into *Agrobacterium tumefaciens* strain GV3101 (pSoup-p19). Agrobacterium suspensions were co-infiltrated into *Nicotiana benthamiana* leaves. After 48 h, infected leaf discs were harvested and ground in 1× Passive Lysis Buffer (PLB). Following centrifugation (10,000 × *g*, 2 min), Firefly luciferase (LUC) activity was measured by mixing 20 µL of supernatant with 100 µL of LAR II. Subsequently, 100 µL of Stop & Glo® Reagent was added to measure the internal control Renilla luciferase (REN) activity. The relative luciferase activity was calculated as the ratio of LUC to REN (RLU1/RLU2). At least three biological replicates were performed, and differences in LUC/REN ratios between effector and empty-vector controls were evaluated using Student's *t*-test.

#### Transient overexpression in F. mandshurica seedlings

To validate regulation in the native system, the coding sequence of *FmNAC104* was cloned into the pROKII vector driven by the CaMV 35S promoter. The construct was transformed into *A. tumefaciens* strain GV3101. One-month-old *F. mandshurica* seedlings were submerged in the bacterial suspension (OD_600_ = 0.6) and subjected to vacuum infiltration. Seedlings infiltrated with the empty pROKII vector served as controls. After 3 d of co-cultivation, samples were harvested for expression analysis.

#### Quantitative real-time PCR (qRT-PCR)

Total RNA was extracted from one-month-old *in vitro* *F. mandshurica* seedlings using a modified CTAB protocol^[92]^. First-strand cDNA was synthesized using the PrimeScript™ RT reagent Kit with gDNA Eraser (Takara) to ensure genomic DNA removal. qRT-PCR was performed using SYBR® Premix Ex Taq™ II (Takara) on a Roche LightCycler® 480 system. Relative expression levels were calculated using the 2^−ΔΔCᴛ^ method^[93]^, with the *FmTubulin* gene serving as the internal reference for normalization. All analyses were conducted with three biological replicates. Results are presented as mean ± SD and were visualized using GraphPad Prism 8. Primer sequences are listed in Supplementary Table S2.

### Prediction of *H. fraxineus* secreted effector candidates

The predicted protein sequences of *H. fraxineus*^[94]^ were first scanned for N-terminal signal peptides using SignalP (v6.0)^[95]^. Proteins with a predicted signal peptide were then analyzed with TMHMM (v2.0)^[96]^ to remove proteins containing transmembrane helices outside the signal peptide region. The remaining secreted, non–membrane-spanning proteins were submitted to EffectorP (v3.0)^[97]^ for effector prediction. Proteins classified as 'likely effectors' were retained as high-confidence effector candidates.

## Results

### Genome sequencing and assembly

To survey the genome of *F. mandshurica*, we first performed a *k*-mer analysis of 104.5 Gb of filtered Illumina reads, which estimated a genome size of ~786.8 Mb and a heterozygosity rate of 1.07% (Supplementary Fig. S1). For *de novo* assembly, we generated 286.9 Gb of polymerase reads on the PacBio Sequel II platform, yielding 16.6 Gb of high-fidelity (HiFi) reads with a mean length of 18.6 kb and > 99.9% accuracy (Table 1). In addition, 70 Gb of Hi-C data and 108 Gb of Illumina whole-genome resequencing reads were produced to support chromosome scaffolding and error correction (Table 1).

**Table 1:** Statistics of the *F. mandshurica* genome assembly.

Category	Statistic	Value
Sequencing data	Sequence platform	PacBio Sequel II, Illumina HiSeq X Ten
	Polymerase read bases (Gb)	286.9
	HiFi read bases (Gb)	16.6
	Hi-C raw data (Gb)	70
	Illumina WGS raw data (Gb)	108
Genome assembly results	Genome size (Mb)	774
	Number of contigs	564
	Contig N50 (Mb)	9.00
	Number of scaffolds (post-Hi-C)	450
	Scaffold N50 (Mb)	34.02
	Chromosomal anchoring rate (%)	97.28
Assembly quality and completeness	BUSCO completeness (Genome) (%)	98.70
	GC content (%)	35.54
	Mean chromosome-level Merqury QV	61.41
	Whole-genome LAI	12.03

Using the HiFi reads, we assembled 564 contigs with a contig N50 of 9.00 Mb (Table 1). Incorporation of the Hi-C data anchored and oriented these contigs into 450 scaffolds, resulting in a chromosome-scale assembly spanning 774 Mb with a scaffold N50 of 34.02 Mb (Table 1). In total, 97.28% of the assembled sequence was placed onto 23 pseudochromosomes, consistent with the karyotype of *F. mandshurica*. The Hi-C contact map showed a strong, continuous main diagonal and clear intrachromosomal interaction blocks, further supporting the accuracy of the chromosomal organization (Fig. 1b). The overall GC content of the assembly was 35.54% (Table 1). Assessment with BUSCO (v5.5.0)^[98,99]^ against the Embryophyta odb10 dataset recovered 98.7% complete conserved genes, indicating a high-quality, near-complete genome assembly. Additional reference-free assembly assessment further supported the high consensus accuracy and repeat-region continuity of the assembly: chromosome-level Merqury analysis yielded a mean QV of 61.41, and the whole-genome LTR Assembly Index (LAI) was 12.03 (Fig. 1c; Table 1).

### Genome annotation

The *F. mandshurica* genome is highly repetitive, with 529.39 Mb of repetitive sequences, corresponding to 67.85% of the assembly (Table 2). Long terminal repeat (LTR) retrotransposons were the most abundant elements: Copia-type and Gypsy-type LTRs occupied 20.77% and 14.15% of the genome, respectively, whereas DNA transposons accounted for 6.61%, LINEs for 1.17%, and SINEs for only 0.01% (Table 2). The chromosomal landscapes of gene density, total repeat density, LTR-Copia, LTR-Gypsy, GC content, and GC skew revealed substantial regional variation in TE accumulation across the 23 chromosomes (Fig. 1a).

**Table 2:** Statistics of *F. mandshurica* genome annotation.

Category	Statistic	Value
Gene annotation	Protein-coding genes	50,092
	Mean gene length (bp)	1,828
	Mean CDS length (bp)	1,201
	BUSCO completeness (Proteins) (%)	96.80
Non-coding RNAs	miRNAs	1129
	snoRNAs	1597
	rRNAs	562
	tRNAs	692
Repeat sequences	Total repetitive sequences (Mb)	529.39
	Repetitive sequence content (%)	67.85
	*LTR-Copia* (%)	20.77
	*LTR-Gypsy* (%)	14.15
	LINE (%)	1.17
	SINE (%)	0.01
	DNA transposons (%)	6.61
Functional annotation	Annotated in at least one database (%)	89.96
	NR (%)	89.87
	eggNOG (%)	80.29
	Pfam (%)	73.14
	COG (%)	75.63
	Swiss-Prot (%)	65.43
	GO (%)	40.20
	KEGG KO (%)	39.44

Combining *ab initio* prediction, homology-based annotation, and transcript evidence, we annotated 50,092 protein-coding genes, with an average gene length of 1,828 bp and an average coding sequence (CDS) length of 1,201 bp (Table 2). BUSCO analysis of the predicted gene set identified 96.8% complete conserved genes, confirming the high completeness of the annotation (Table 2). Functional annotations were assigned to 45,062 genes (89.96%) based on similarity searches against public databases (Table 2). Database-specific annotation rates were further summarized in Table 2, including NR (45,020 genes, 89.87%), eggNOG (40,220 genes, 80.29%), COG (37,885 genes, 75.63%), Pfam (36,637 genes, 73.14%), Swiss-Prot (32,775 genes, 65.43%), GO (20,137 genes, 40.20%), KEGG KO (19,757 genes, 39.44%), and KEGG pathway annotations (12,486 genes, 24.93%).

In addition to protein-coding genes, we identified 3,980 noncoding RNA (ncRNA) genes, including 1,129 microRNAs (miRNAs), 1,597 small nucleolar RNAs (snoRNAs), 562 ribosomal RNAs (rRNAs), and 692 transfer RNAs (tRNAs) (Table 2), indicating a complex repertoire of regulatory and structural ncRNAs in the *F. mandshurica* genome.

### Phylogenetic position and divergence history of *F. mandshurica*

To place *F. mandshurica* in an evolutionary context, we reconstructed a species phylogeny using single-copy orthologs from twelve representative angiosperms. After stringent filtering for alignment completeness and sequence length homogeneity, 151 high-confidence single-copy orthologs were retained for concatenated maximum-likelihood analysis. This conservative strategy produced a well-resolved phylogeny with uniformly high support for major nodes (Fig. 2a).

**Figure 2:**
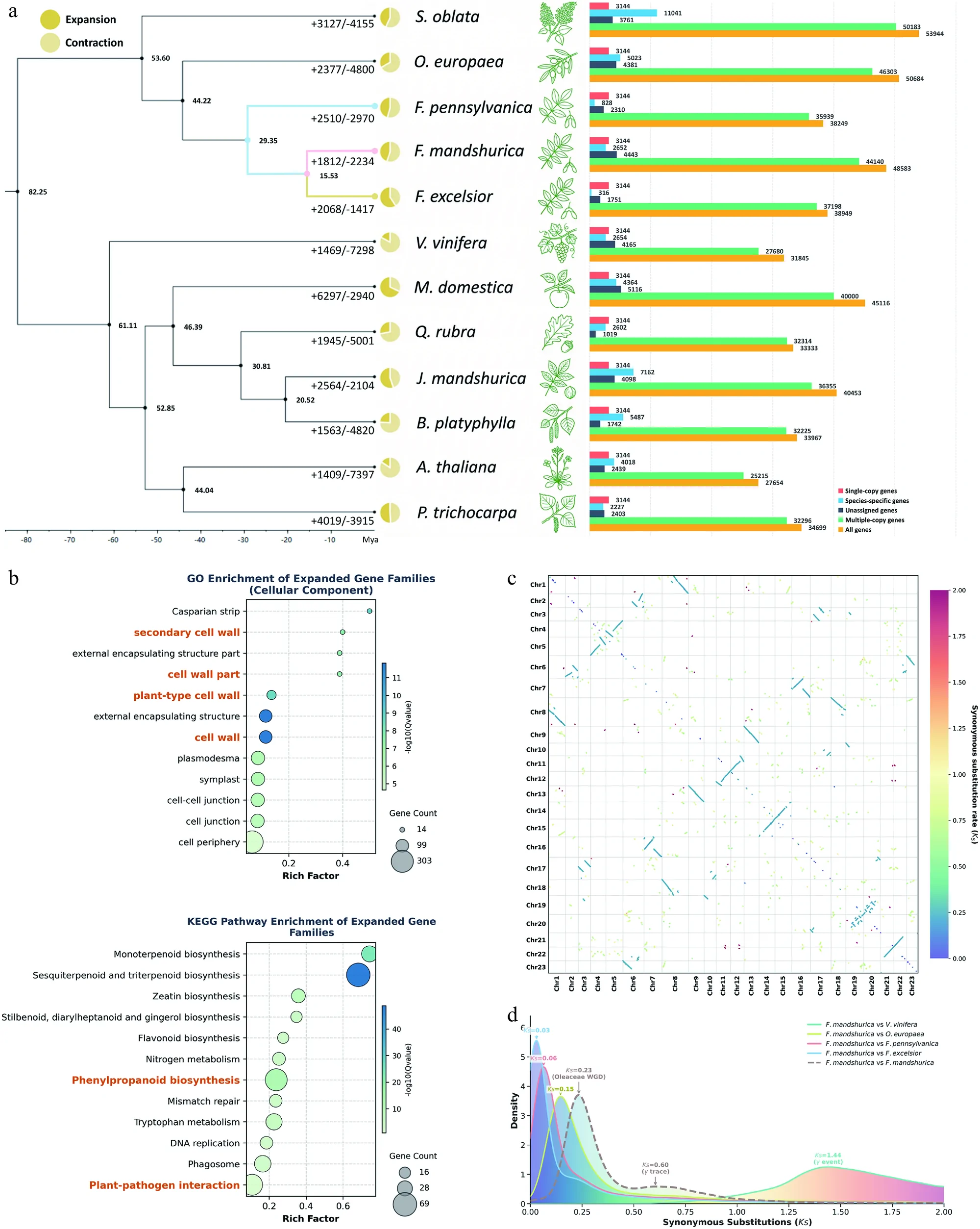
Phylogeny, gene family evolution, and ancient WGD in *Fraxinus mandshurica*. (a) Time-calibrated phylogeny of *F. mandshurica* and 11 other angiosperms based on 151 single-copy orthologs. Node labels indicate estimated divergence times (Mya), and numbers beside terminal branches indicate expanded (+) and contracted (−) gene families. Bar plots show the proportions of single-copy, multi-copy, and species-specific genes. (b) GO and KEGG enrichment of expanded gene families in *F. mandshurica*, highlighting terms and pathways related to cell wall biogenesis and plant defense. Bubble size represents gene count, and color indicates significance (−log_10_ *Q*-value). (c) Intra-genomic synteny dot-plot of the 23 chromosomes. The color gradient reflects the median *K_s_* of collinear blocks, ranging from blue/cyan (younger duplications, low *K_s_*) to orange/red (ancient duplications, high *K_s_*). (d) *K_s_* distributions of syntenic gene pairs. The dashed line represents *F. mandshurica* paralogs (self-alignment), while solid lines represent orthologs between *F. mandshurica* and other species. Key peaks are marked: the Oleaceae-wide WGD (O-*α*, *K_s_* ≈ 0.23), the divergence from *O. europaea* (*K_s_* ≈ 0.15), and the ancestral eudicot *γ* triplication traces (*K_s_* ≈ 0.60 in self-alignment; *K_s_* ≈ 1.44 vs *V. vinifera*).

The resulting tree confirms that *F. mandshurica* belongs to the Oleaceae family and forms a strongly supported clade with other *Fraxinus* species, *Syringa oblata*, and *Olea europaea* (Fig. 2a). Molecular dating, calibrated with fossil constraints, indicated a relatively recent radiation within the sampled ashes: the Eurasian *Fraxinus* clade (*F. mandshurica* and *F. excelsior*) diverged from the North American lineage (*F. pennsylvanica*) ~29.4 million years ago (Mya), whereas *F. mandshurica* and *F. excelsior* separated more recently, at ~15.5 Mya (Fig. 2a). This temporal framework provides a robust basis for interpreting lineage-associated genomic changes associated with the distinct ecological niches of these ash species.

### Lineage-associated gene family expansions point to adaptations in cell wall biogenesis and defense

To identify candidate genomic changes underlying the distinctive traits of *F. mandshurica*, we examined gene family turnover across the twelve species using CAFE. Along the terminal branch leading to *F. mandshurica*, 1,812 gene families were significantly expanded and 2,234 were contracted (Fig. 2a).

We next assessed the functional implications of these expansions. Gene Ontology (GO) and KEGG enrichment analyses of the expanded families converged on two biological themes central to this study: cell wall modification and plant defense (Fig. 2b). In the Cellular Component category, expanded families were significantly enriched for terms defining structural barriers, including 'secondary cell wall', 'plant-type cell wall', 'external encapsulating structure', 'Casparian strip', and 'plasmodesma' (Fig. 2b). These enrichments provide a genomic footprint for the highly specialized wood properties and physical defense barriers of *F. mandshurica*.

In parallel, genes in expanded families were overrepresented in KEGG pathways associated with plant defense and specialized metabolism. Top enriched pathways included 'Monoterpenoid biosynthesis', 'Sesquiterpenoid and triterpenoid biosynthesis', 'Phenylpropanoid biosynthesis', and 'plant–pathogen interaction' (Fig. 2b). Notably, several transcription factor and enzyme families with dual roles in development and stress responses, such as NAC transcription factors and class III peroxidases (*PRX*s), were represented among the expanded gene families in the CAFE analysis. Collectively, these patterns indicate that a substantial fraction of lineage-associated gene-family gains is concentrated in networks that can synergistically reinforce both secondary cell wall formation and defensive capacity.

### Ancient whole-genome duplications shaped the *F. mandshurica* genome architecture

To reconstruct the polyploidization history of *F. mandshurica*, we first performed a comprehensive intra-genomic synteny analysis. The self-alignment dot-plot revealed extensive collinear paralogous blocks distributed across the 23 chromosomes, providing robust structural evidence for large-scale duplication events (Fig. 2c). These syntenic blocks exhibit a clear temporal hierarchy based on their synonymous substitution rates (*K_s_*). The structurally well-preserved chromosomal segments—highlighted in the blue/cyan range—correspond to relatively recent duplication events. In contrast, the more fragmented blocks, visualized in the orange/red spectrum, represent the distinctive genomic footprints of ancient polyploidization (Fig. 2c).

To precisely resolve the timing of these events, we analyzed the *K_s_* distribution of syntenic gene pairs (Fig. 2d). The density curve displayed two prominent peaks indicative of successive whole-genome duplications (WGDs). The primary peak, centered at *K_s_* ≈ 0.23, corresponds to the extensive blue/cyan syntenic blocks. Crucially, this peak is situated to the right of the speciation peak identified between *F. mandshurica* and *Olea europaea* (*K_s_* ≈ 0.15). This relative chronology (*K_s_* WGD > *K_s_* Speciation) supports the inference that this duplication event occurred prior to the divergence of the *Fraxineae* and *Olea* lineages, confirming it as a shared Oleaceae-specific WGD (the O-*α* event).

Furthermore, we identified the remnants of the ancestral core eudicot *γ* (gamma) triplication. Intriguingly, the *K_s_* peak corresponding to this ancient event in the *F. mandshurica* self-alignment (*K_s_* ≈ 0.60) appears at a significantly lower value than the divergence peak between *F. mandshurica* and the core eudicot reference *Vitis vinifera* (*K_s_* ≈ 1.44) (Fig. 2d). Theoretically, since the *γ* event occurred shortly before the diversification of core eudicots, these two peaks typically overlap in species with standard evolutionary rates. However, the distinct 'left-shift' of the *γ* duplication peak in *F. mandshurica* suggests a significantly slower molecular evolutionary rate in the *Fraxinus* lineage compared to *Vitis*. This slower molecular evolution is likely attributable to the long generation time and woody perennial life history of ash trees, which results in a slower accumulation of synonymous substitutions over millions of years compared to herbaceous or vine lineages.

### Comparative genomics reveals structural variations and defense-related gene expansions associated with the enhanced disease tolerance of *F. mandshurica*

To investigate the genomic basis for the divergent adaptive traits among ash species, particularly the enhanced disease tolerance of *F. mandshurica*, we first assessed its constitutive basal defense capacity. While *F. mandshurica* is known to tolerate the ash dieback pathogen (*H. fraxineus*), it remains unclear whether this resilience represents a specific response or a heightened basal immunity. To test this, we conducted inoculation assays using two broad-spectrum fungal pathogens, *Fusarium iranicum* and *Colletotrichum sojae*, against the resistant *F. mandshurica* and the susceptible European ash (*F. excelsior*).

Quantitative analysis across nine biological replicates revealed a striking contrast in host response. *F. mandshurica* exhibited enhanced broad-spectrum disease tolerance, restricting fungal colonization to small, contained spots. In contrast, *F. excelsior* leaves developed severe, spreading necrotic lesions (Fig. 3a). Statistical quantification confirmed that the lesion areas in *F. mandshurica* were significantly smaller than those in *F. excelsior* (Fig. 3b; *p* < 0.01; Supplementary Dataset S2). This result indicates that *F. mandshurica* possesses a stronger basal leaf defense capacity against these two fungal pathogens than *F. excelsior*, providing a phenotypic anchor for our subsequent genomic analyses.

**Figure 3:**
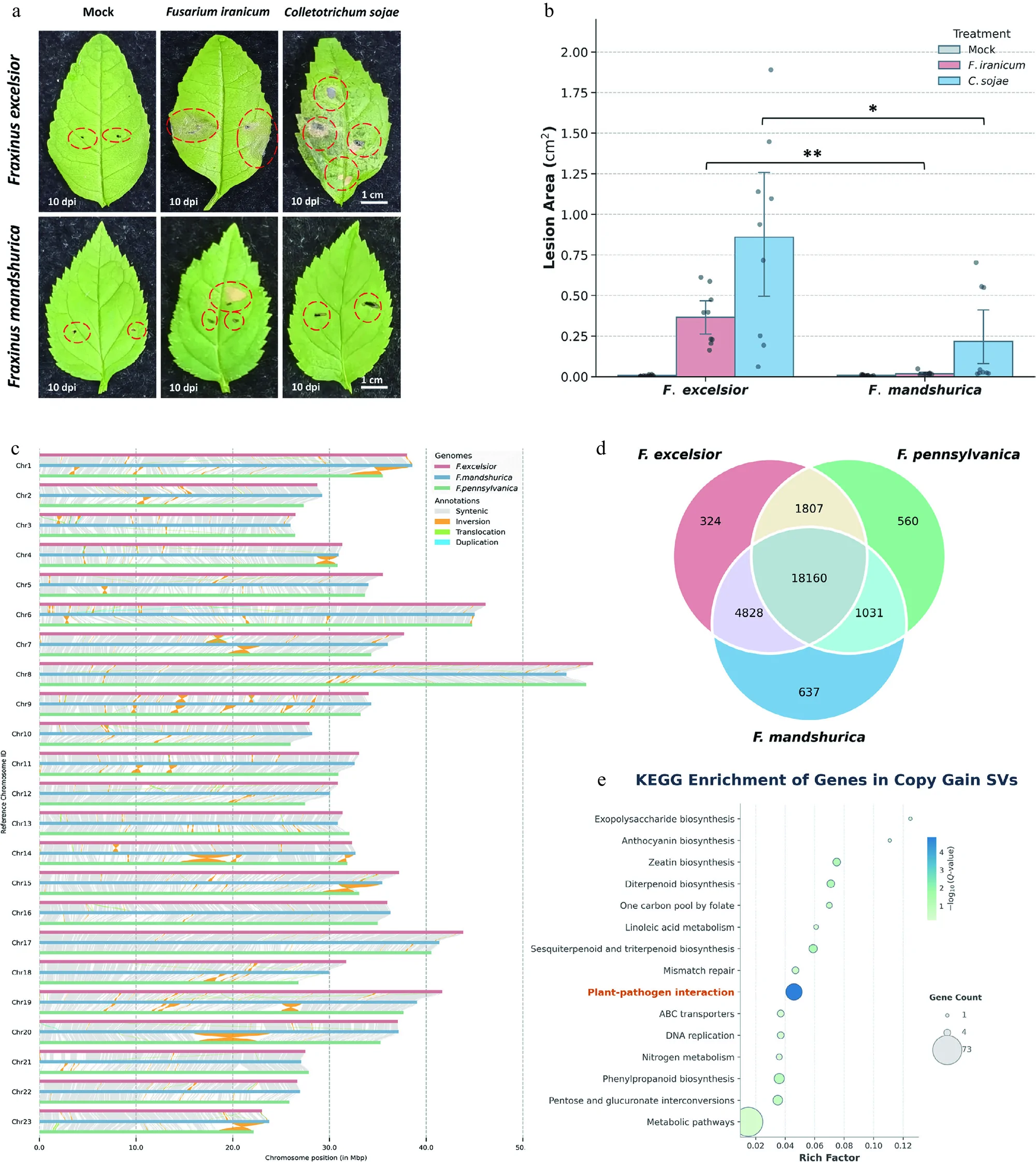
Comparative genomics and defense-related structural variations in *F. mandshurica*. (a) Broad-spectrum disease resistance phenotypes. Leaves of *F. excelsior* (susceptible) and *F. mandshurica* (resistant) were inoculated with *Fusarium iranicum* and *Colletotrichum sojae.* (b) Quantitative analysis showed significantly smaller lesion areas in *F. mandshurica than in F. excelsior.* Data represent mean ± SD (*n* = 9); asterisks indicate significant differences (Student's *t*-test, * *p* < 0.05, ** *p* < 0.01). (c) Chromosome-level structural variation survey comparing *F. mandshurica* (center, blue bars) with *F. excelsior* (top, red bars) and *F. pennsylvanica* (bottom, green bars). Symbols indicate inversions, translocations, and duplications detected along homologous chromosomes. (d) Venn diagram showing the overlap of orthologous gene families among the three *Fraxinus* species. The core genome (center) and lineage-specific clusters are indicated. (e) KEGG pathway enrichment of genes located in Copy Gain structural variation (SV) regions in *F. mandshurica*. The 'plant–pathogen interaction' pathway is significantly enriched, suggesting a genomic basis for enhanced immunity. Bubble size indicates gene count, and color intensity represents significance (−log_10_ *Q*-value).

To unravel the genetic architecture supporting this enhanced defense phenotype, we extended our analysis to a chromosome-level comparative survey against *F. excelsior* and the North American green ash (*F. pennsylvanica*). Because *F. pennsylvanica* was not included in our inoculation assays, it was used here as a phylogenetically divergent genomic reference rather than as an experimentally phenotyped species. Previous ash dieback studies have described *F. pennsylvanica* as less susceptible than *F. excelsior* but not fully resistant, showing moderate or mild symptoms under field or inoculation conditions^[100−102]^. Chromosome-level synteny analysis revealed substantial collinearity between the two Eurasian species (*F. mandshurica* and *F. excelsior*). In contrast, comparisons with *F. pennsylvanica* uncovered numerous large-scale chromosomal rearrangements. The synteny plot highlights multiple structural variation events, including inversions, translocations, and duplications, between *F. mandshurica* and *F. pennsylvanica* (Fig. 3c). These structural variations underscore the genomic divergence resulting from geographic isolation.

We further examined gene family conservation using a Venn diagram (Fig. 3d). While 18,160 orthogroups were shared among the three species (representing the core *Fraxinus* genome), *F. mandshurica* shared considerably more orthogroups with *F. excelsior* (4,828) than with the phylogenetically distant *F. pennsylvanica* (1,031). Notably, *F. mandshurica* possessed 637 species-specific gene families, potentially harboring lineage-associated adaptive factors (Fig. 3d).

Finally, we focused on 'Copy Gain' structural variations (SVs)—regions where gene copy numbers have expanded in *F. mandshurica* relative to its relatives—to pinpoint candidate defense-related genes. KEGG enrichment analysis of genes located in these Copy Gain regions revealed a pronounced bias toward defense- and metabolism-related pathways (Fig. 3e). Strikingly, 'plant–pathogen interaction' was the top enriched pathway, followed by 'Diterpenoid biosynthesis' and 'Phenylpropanoid biosynthesis' (Fig. 3e). The specific expansion of these gene repertoires via structural variation provides a plausible genomic basis for the enhanced constitutive defense observed in *F. mandshurica*.

### Tandem and proximal duplications enrich candidate positively selected defense-related genes

While the identification of Copy Gain regions provides a snapshot of genomic expansion, we sought to investigate the duplication modes contributing to defense-related gene diversification. We classified all duplicated genes in the *F. mandshurica* genome into five categories based on their genomic context: whole-genome (WGD), tandem (TD), proximal (PD), transposed (TRD), and dispersed (DD) duplications (Fig. 4a). Although WGD and DD account for the largest absolute numbers of duplicated genes (16,926 and 17,345, respectively), they showed relatively low proportions of candidate positively selected genes, with 0.2% and 4.5% of genes in these categories showing signatures consistent with positive selection (*K_a_/K_s_* > 1) (Fig. 4a).

**Figure 4:**
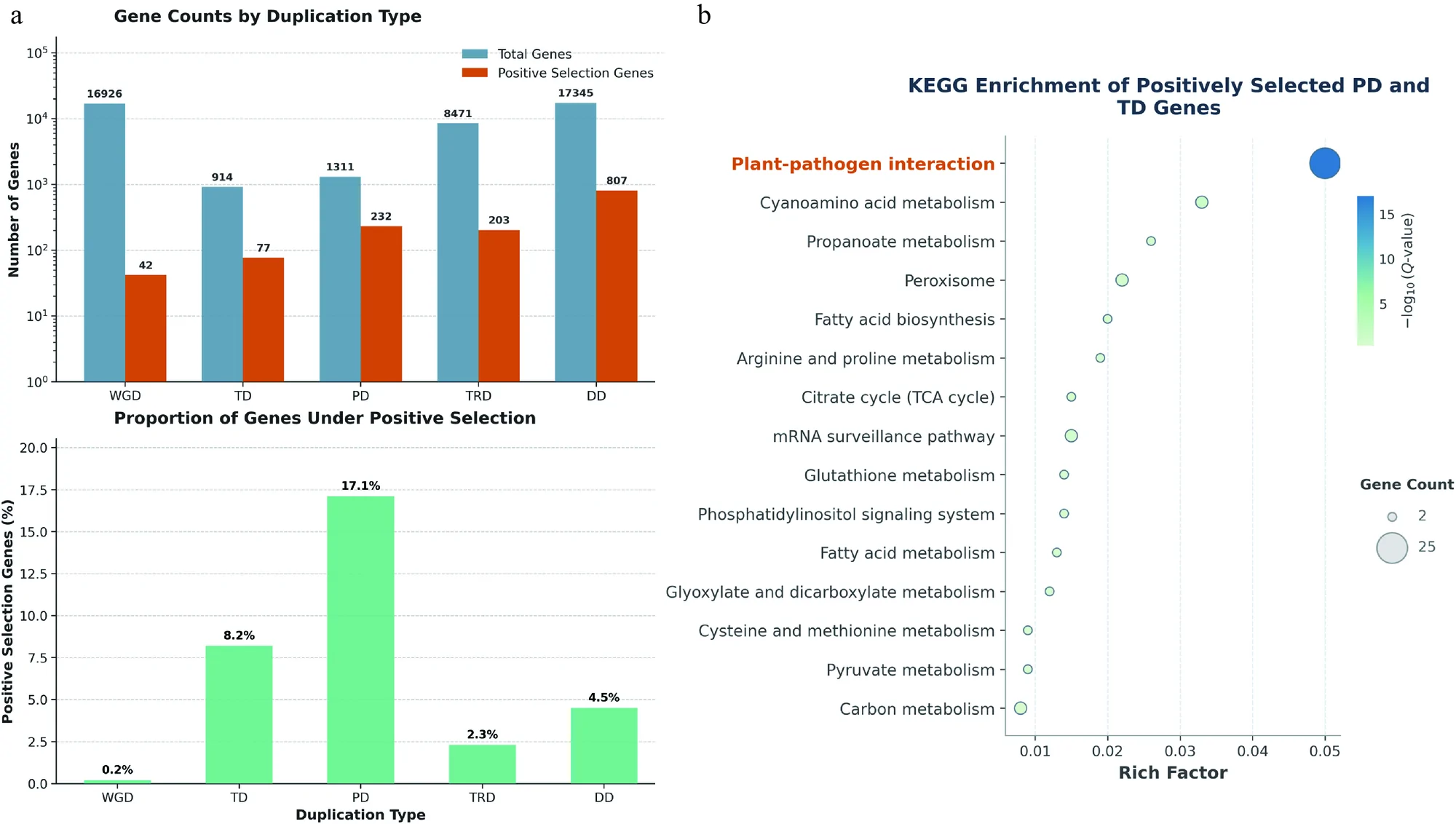
Local duplications under positive selection enrich for defense pathways in *F. mandshurica.* (a) Gene duplication landscape and selection pressure. Top panel: Total number of genes (blue bars) and genes under positive selection (*K_a_/K_s_* > 1; orange bars) classified by duplication mode: WGD (whole-genome), TD (tandem), PD (proximal), TRD (transposed), and DD (dispersed). Bottom panel: The proportion (%) of positively selected genes within each duplication category. PD and TD duplicates showed the highest proportions of candidate positively selected genes, at 17.1% and 8.2%, respectively. (b) KEGG pathways enriched in positively selected genes derived from TD and PD events. The 'plant–pathogen interaction' pathway shows the most significant enrichment, suggesting a potential contribution of local duplications to defense-related gene diversification. Bubble size represents gene number, and color intensity indicates significance (−log_10_ *Q*-value).

In contrast, tandem (TD) and proximal (PD) duplications, which arise from local, small-scale replication events, were enriched for candidate positively selected genes. Despite representing a smaller fraction of the total gene count, genes originating from TD and PD events exhibited remarkably higher proportions under positive selection, reaching 8.2% and 17.1%, respectively (Fig. 4a). This finding indicates that these localized duplication modes may provide important substrates for functional diversification and adaptive divergence.

To connect these evolutionary dynamics with the functional traits of *F. mandshurica*, we performed KEGG enrichment analysis specifically on the positively selected genes derived from TD and PD events. The analysis revealed significant enrichment of the 'plant–pathogen interaction' pathway, which exhibited the highest significance level (Fig. 4b). Other enriched pathways, such as 'Cyanoamino acid metabolism' and 'Peroxisome', further point to roles in defense-related secondary metabolism and stress response. These findings suggest that localized duplications followed by positive selection may have contributed to the diversification of immune- and stress-related gene networks in *F. mandshurica*.

### An expanded and dynamically evolving NLR repertoire is associated with enhanced disease tolerance in *F. mandshurica*

Our duplication and selection analyses highlighted that tandem (TD) and proximal (PD) duplicates are hotspots of positive selection, predominantly enriching the 'plant–pathogen interaction' pathway. Many defense-related candidates were associated with the nucleotide-binding leucine-rich repeat (NLR) family, which constitutes the core of the plant intracellular immune system. After NLR-Annotator prediction and conserved-domain verification, the retained candidates were classified based on the presence of the essential NB-ARC domain (Supplementary Dataset S3).

To place this NLR repertoire variation in a broader evolutionary context, we compared NLR repertoires across ten *Fraxinus* species. The resulting phylogeny and gene counts revealed a highly dynamic history of NLR gain and loss (Fig. 5a). In *F. mandshurica*, 353 NLR genes were identified, and these were further represented by 156 NLR orthogroups and 32 species-specific NLRs in the cross-species comparison shown in Fig. 5a. Accordingly, *F. mandshurica* harbors one of the largest NLR repertoires among the sampled *Fraxinus* species, whereas species such as *F. sogdiana* show a substantially smaller overall NLR profile. Because Fig. 5a summarizes three related count classes (identified NLR genes, NLR orthogroups, and species-specific NLRs), these interspecific differences should be interpreted as evidence of lineage-associated repertoire expansion and diversification, rather than as a direct proxy for realized disease resistance. These interspecific differences suggest that NLR gain/loss and lineage-specific diversification may partly contribute to variation in immune surveillance capacity among *Fraxinus* species, although NLR copy number alone is unlikely to fully determine realized disease resistance.

**Figure 5:**
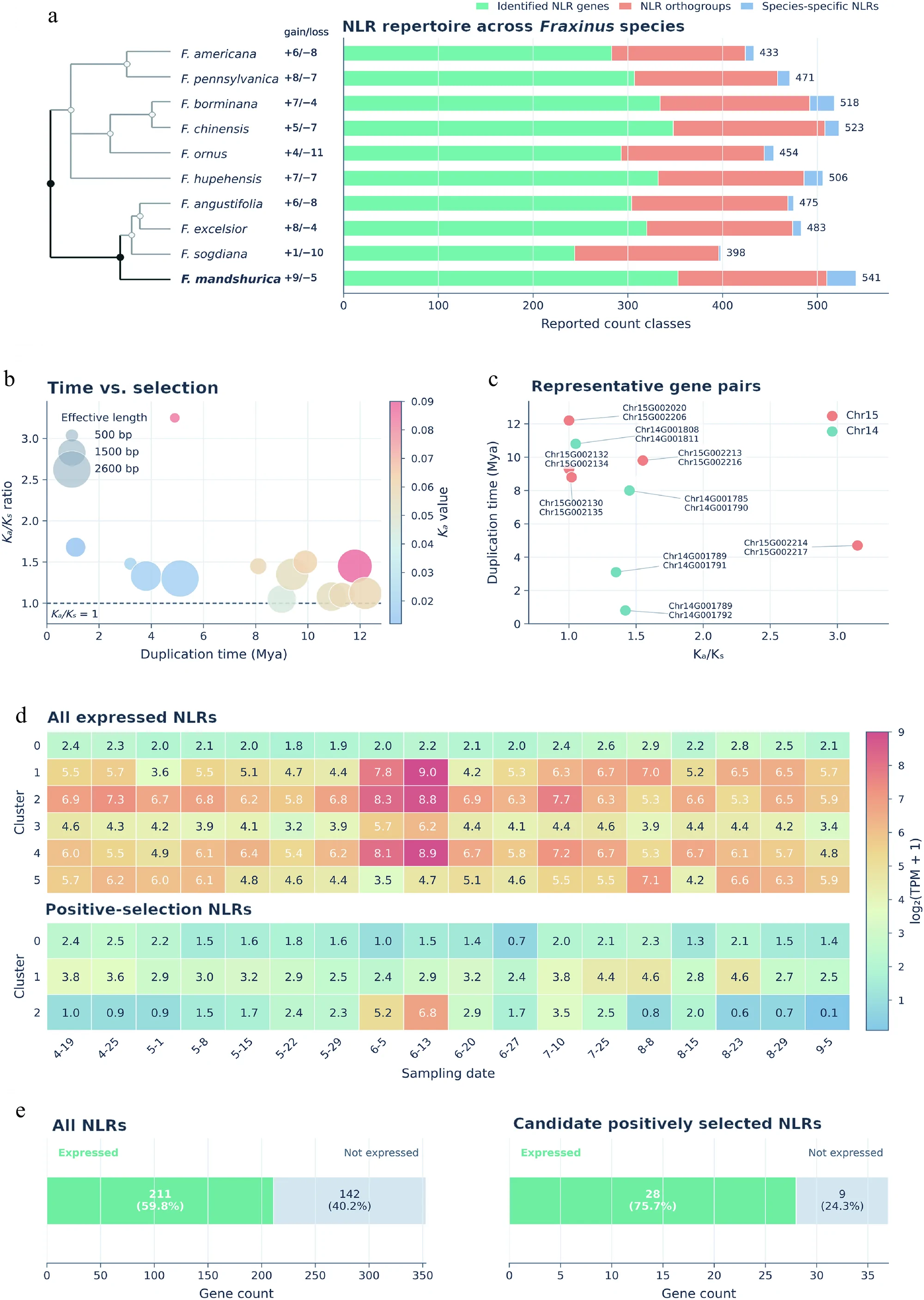
Expansion, adaptive evolution, and functional deployment of NLR genes in *F. mandshurica*. (a) Evolutionary dynamics of NLR repertoires across ten Fraxinus species. The phylogeny (left) shows inferred gene family gains (+) and losses (−) along each branch. The stacked bars (right) summarize three count classes: identified NLR genes, NLR orthogroups, and species-specific NLRs; the number at the end of each bar indicates the total reported count across these three classes. *F. mandshurica* exhibits one of the largest overall NLR profiles among the sampled species. (b) Relationship between duplication time and selective pressure among NLR paralogs. Each bubble represents an NLR gene pair; the x-axis indicates duplication time (Mya), the y-axis indicates *K_a_/K_s_* ratio, bubble size represents effective length, bubble color represents *K_a_* value, and the dashed horizontal line marks *K_a_/K_s_* = 1. (c) Representative candidate positively selected NLR gene pairs on chromosomes 14 and 15. The scatter plot shows duplication time vs *K_a_/K_s_* ratio, with point color indicating chromosome identity and labels denoting representative gene pairs. (d) Temporal expression heatmaps of NLR genes during xylem development. The upper panel shows all 211 expressed NLRs grouped into six clusters, and the lower panel shows 28 expressed candidate positively selected NLRs grouped into three clusters. Expression values are shown as log_2_(TPM + 1). (e) Expression status of the total NLR set and the candidate positively selected subset. Stacked horizontal bar charts show the proportions of expressed and not expressed genes for all NLRs (211 expressed, 142 not expressed) and candidate positively selected NLRs (28 expressed, nine not expressed), respectively.

We next examined the evolutionary forces associated with this NLR repertoire. *K_a_/K_s_* analysis of NLR paralogs identified numerous loci under strong positive selection (*K_a_/K_s_* > 1). By plotting *K_a_/K_s_* ratios against duplication times, we found that many positively selected NLR pairs arose recently, mostly within the last ~12 million years (Fig. 5b). Detailed analysis of specific gene pairs highlighted several very recent duplicates, particularly on chromosomes 14 and 15, spanning duplication times of approximately 1–12 Mya and exhibiting elevated *K_a_/K_s_* ratios (Fig. 5c; Supplementary Dataset S3). These findings are consistent with recurrent diversification of NLR paralogs under pathogen-mediated selection pressure.

To evaluate the transcriptional deployment of this NLR repertoire, we quantified the transcript levels of all 353 NLR genes across the xylem developmental time-series. Overall, 59.8% (211/353) were expressed in at least one developmental stage. Strikingly, this proportion increased to 75.7% (28/37) among the positively selected NLR subset (Fig. 5e). This observation suggests that candidate positively selected NLRs are more likely to be transcriptionally active in developing xylem than the overall NLR set.

We further explored the temporal regulation of these immune receptors using hierarchical clustering. The 211 expressed NLRs segregated into six co-expression modules with distinct seasonal profiles (Fig. 5d, upper panel). Several large clusters (e.g., Clusters 1, 2, and 4) displayed coordinated expression peaks from early to mid-summer (June 5–20), coinciding with the Transition Stage, a phase of rapid xylem expansion and heightened tissue vulnerability. Focusing specifically on the positively selected NLRs revealed more specialized temporal partitioning: the 28 expressed candidate positively selected NLRs formed three clusters (Fig. 5d, lower panel). While one module mirrored the mid-season peak of the global set, another exhibited a pronounced late-season activation (July 25–August 29). This temporal layering suggests a staged immune-surveillance pattern, in which broadly expressed NLRs may contribute to defense during the Transition Stage, whereas a subset of recently duplicated or candidate positively selected NLRs may remain active during later wood maturation and lignification.

### Transcriptional architecture of wood formation: linking structural and defense-related programs

Having characterized the rapidly expanded and dynamically evolving NLR repertoire of *F. mandshurica*, we next investigated wood formation and structural reinforcement, another key component of its adaptive phenotype. We asked whether immune-related programs and secondary cell wall formation are transcriptionally coordinated during seasonal xylem development. Our gene family and pathway enrichment analyses provided an initial clue, revealing enrichment patterns associated with plant defense and secondary cell wall biogenesis. In particular, the phenylpropanoid biosynthesis pathway emerged as a central metabolic hub, supplying precursors both for lignin, the major load-bearing component of the secondary cell wall, and for phenylpropanoid-derived defense metabolites.

These observations led us to hypothesize that the temporal regulation of this dual-function pathway during wood development may contribute to a coordinated balance between mechanical strengthening and constitutive defense. To examine this hypothesis, we analyzed the high-resolution seasonal time-series RNA-seq dataset from developing xylem.

### Stage-specific regulatory networks reveal transcriptional programs during wood development

We first analyzed transcription-factor dynamics across the 18-point developing-xylem time series (Supplementary Fig. S3). The transcriptomic analysis identified a core set of 131 transcription factors (TFs) exhibiting significant temporal modulation (FDR < 0.05; |log_2_ fold change| > 1), suggesting dynamic stage-associated regulation. To resolve their hierarchical deployment, we applied the Time-Ordered Gene Co-expression Network (TO-GCN) algorithm, which grouped these TFs into six temporal modules (L1–L6), representing distinct transcriptional waves across the growing season. Modules L1–L3 were associated with early-season xylem activity, Module L4 (33 TFs; 25.2%) peaked during the Transition Stage, and Modules L5 (26 TFs; 19.8%) and L6 (10 TFs; 7.6%) were activated sequentially from mid- to late summer, reflecting a progressive transition from cambial activation to intensive secondary wall biosynthesis (Fig. 6a; Supplementary Dataset S4).

**Figure 6:**
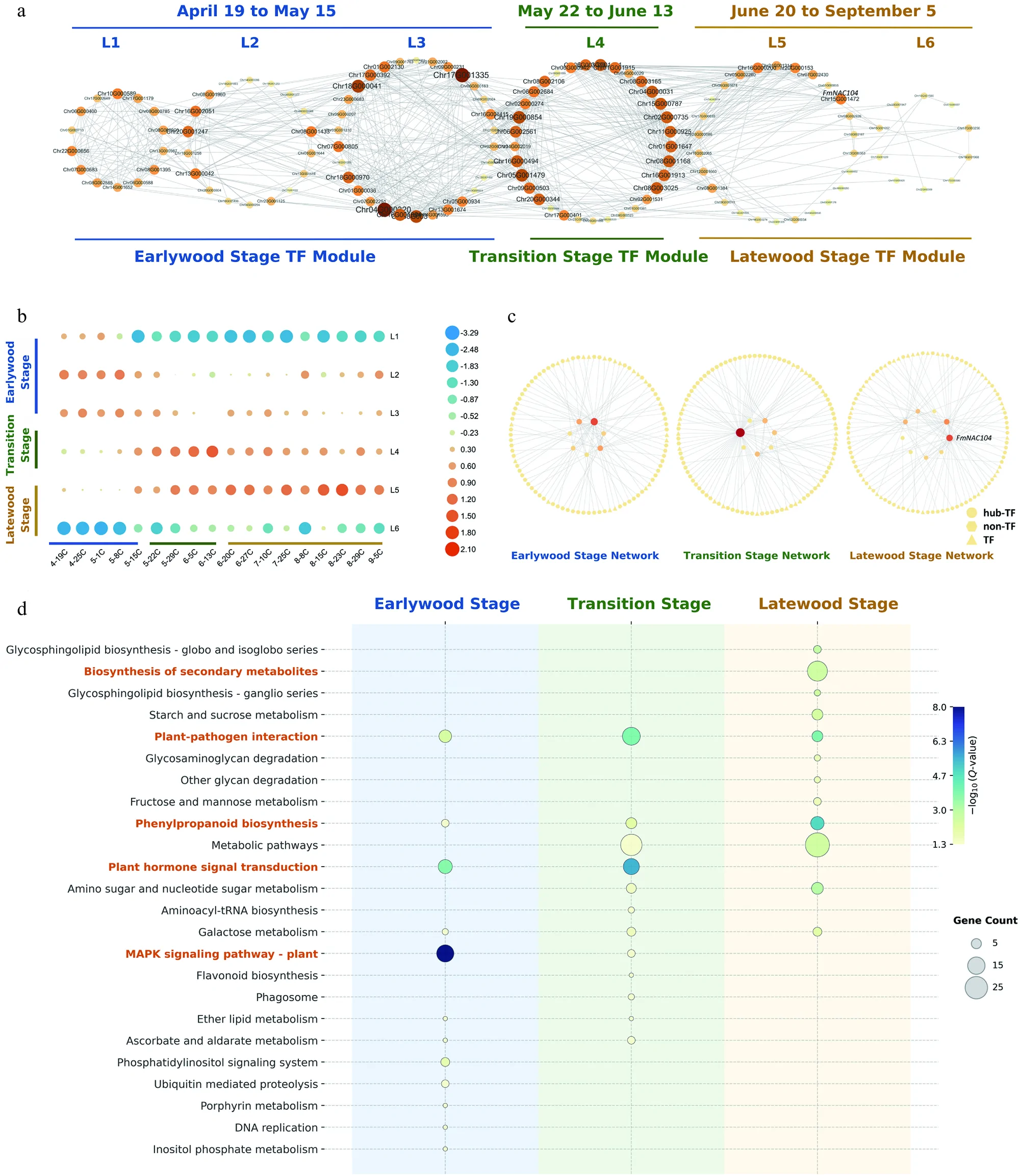
Stage-resolved transcription factor networks controlling wood development in *F. mandshurica*. (a) Time-ordered TF co-expression network (TO-GCN) showing six temporal modules (L1–L6) aligned with the three developmental phases: Earlywood Stage, Transition Stage, and Latewood Stage. (b) Mean expression profiles of each TF module across sampling dates, defining the distinct physiological stages of wood formation. (c) Directed regulatory networks constructed via GENIE3 for each stage. Central nodes represent hub TFs (larger nodes indicate higher connectivity), and peripheral nodes represent downstream targets. Note the prominent hub *FmNAC104* (red node) in the Latewood network. (d) KEGG pathway enrichment of TF targets at each stage, illustrating a functional shift from signaling-dominated programs in Earlywood/Transition to phenylpropanoid and secondary metabolism in Latewood. Bubble size indicates gene count, and color intensity represents significance (−log_1__0_ *Q*-value).

Integrating these transcriptional patterns with field phenology and anatomical observations^[103]^, we delineated three biologically interpretable phases: an Earlywood Stage (April 19–May 15) characterized by the onset of cambial reactivation and initial vessel differentiation; a Transition Stage (May 22–June 13) marked by rapid cell expansion and the shift from conductive to support tissue; and a Latewood Stage (June 20–September 5) defined by the deposition of dense, highly lignified fibers (Fig. 6b).

To infer the biological processes orchestrated at each stage, we utilized the GENIE3 algorithm to construct directed regulatory networks (Fig. 6c). KEGG enrichment analysis of the downstream targets revealed a dynamic functional transition across the growing season. The Earlywood Stage was associated with targets enriched for 'MAPK signaling' and 'Plant hormone signal transduction', consistent with signaling programs involved in cambial reactivation and early xylem development (Supplementary Dataset S5).

As development progressed into the Transition Stage, we observed significant enrichment of the 'plant–pathogen interaction' pathway (Fig. 6d). Notably, this enrichment coincided with rapid tissue expansion and the increased transcriptional activity of expressed NLR genes described earlier (Fig. 5d), suggesting a coordinated deployment of immune surveillance during a developmentally vulnerable growth phase.

Moving into the Latewood Stage, the regulatory landscape underwent a marked shift toward secondary metabolism and cell wall-associated pathways. While signaling pathways persisted, the 'Phenylpropanoid biosynthesis' pathway exhibited a progressive enrichment trajectory—initiating at low levels in earlier stages and culminating in a peak during latewood formation (Fig. 6d). This shift, coupled with the enrichment of 'Biosynthesis of secondary metabolites', reflects the intensified demand for lignin deposition and the accumulation of defense-related metabolites. Topological inspection of the Latewood network identified *FmNAC104* as a highly connected hub within the L5 module (Fig. 6c, Latewood Stage Network red node). Its centrality in this specific window, regulating a network biased toward both structural lignification and secondary metabolism, highlighted *FmNAC104* as a strong candidate hub potentially connecting latewood lignification with defense-related secondary metabolism.

### Identification and validation of a latewood-recruited FmNAC104–FmPRX1 regulatory module

Our transcriptomic blueprint revealed the core regulatory logic of wood formation, yet the specific evolutionary innovations driving the superior wood properties of *F. mandshurica* remained elusive. Compared to *Populus trichocarpa*—a model diffuse-porous hardwood with relatively lower wood density—*F. mandshurica* (a ring-porous species) exhibits exceptional mechanical strength and distinct lignification patterns. We hypothesized that lineage-specific gene family expansions, particularly of transcription factors (TFs) active during the crucial Latewood Stage, underpin these distinct traits.

To test this, we employed a stepwise filtering strategy to identify candidate latewood-associated regulators. First, we intersected the set of *F. mandshurica* lineage-expanded gene families with our stage-specific transcriptomic profiles. This analysis narrowed down the candidates to a concise list of seven 'high-priority' TFs that are both evolutionarily expanded and transcriptionally upregulated during latewood formation (Fig. 7a, top; Supplementary Table S3). Among them, *FmNAC104* emerged as a top candidate, given the established role of NACs in secondary wall deposition, alongside two DOF factors (*FmDOF2.4* and *FmDOF5.1*). To address the evolutionary status of this candidate module more precisely, we further performed formal NAC and PRX family identification using Pfam/HMMER domain searches. This analysis identified 224, 208, 200, 212, and 155 high-confidence NAC genes, and 126, 123, 113, 135, and 108 high-confidence PRX genes in *F. mandshurica*, *F. excelsior*, *F. sogdiana*, *F. pennsylvanica*, and *P. trichocarpa*, respectively. Domain-validation results, mapped anchors, exact copy numbers, and orthogroup members are provided in Supplementary Dataset S1.

**Figure 7:**
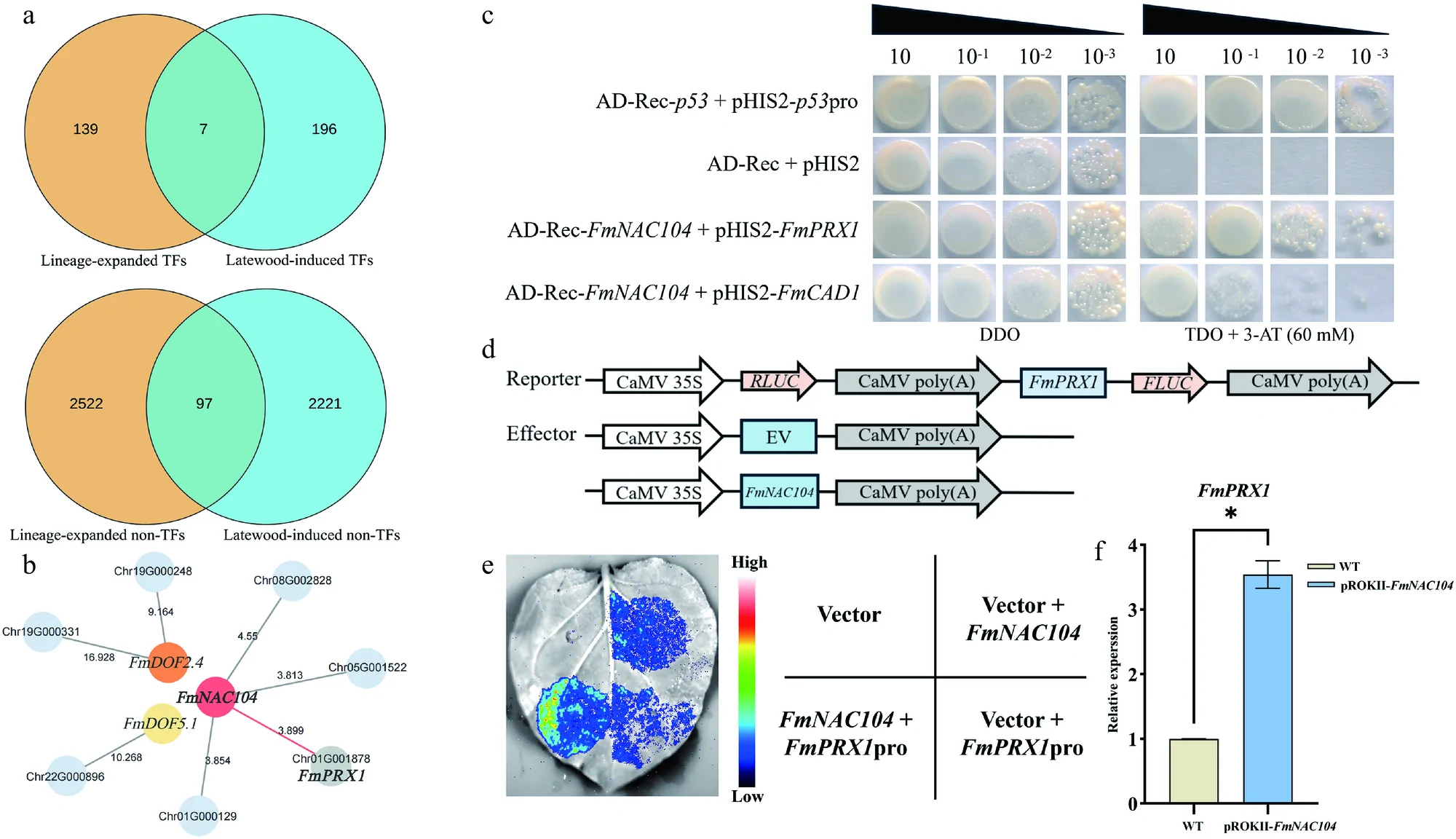
A latewood-recruited FmNAC104–FmPRX1 regulatory module in *F. mandshurica*. (a) Identification of high-priority candidates. Top Venn diagram: Intersection of lineage-expanded TFs and Latewood-induced TFs yields seven candidates (including *FmNAC104*). Bottom Venn diagram: Intersection strategy for identifying expanded, Latewood-induced target genes. (b) High-confidence regulatory sub-network integrating GENIE3 weights and motif analysis. The network highlights the interaction between *FmNAC104* (red node) and *FmPRX1* (target), alongside other candidates like *FmDOF2.4*. (c) Yeast one-hybrid (Y1H) assay showing specific binding of FmNAC104 to the *FmPRX1* promoter (*pFmPRX1*) but not to the negative control promoter (*pFmCAD1*) or empty vectors on selective media (TDO/3-AT). (d) Schematic of the dual-luciferase reporter constructs used to test transcriptional activation. (e) Dual-luciferase assay in *Nicotiana benthamiana* leaves showing strong activation of *pFmPRX1* by FmNAC104 (LUC signal). (f) *In planta* validation. qRT-PCR analysis shows the relative expression of endogenous *FmPRX1* in *F. mandshurica* seedlings transiently overexpressing *FmNAC104* (pROKII-*FmNAC104*) compared with empty-vector controls (EV). Data represent the mean ± SD (*n* = 3); asterisk indicates significance (Student's *t*-test, *p* < 0.05).

To rigorously identify the downstream targets of these regulators, we developed an integrated scoring metric that synergizes regulatory strength with physical binding potential. This metric calculates an Integrated Confidence Score by multiplying the GENIE3 weight (functional association) by a motif enhancement factor (Motif Count + 1), prioritizing targets that are both co-expressed and possess specific binding sites (e.g., predicted NAC binding motifs in their promoter regions). Applying this filter to identify targets that are also lineage-expanded and latewood-induced (Fig. 7a, bottom), we reconstructed a high-confidence sub-network (Fig. 7b; Supplementary Table S4). Among the top-scoring candidates, the regulation of *FmPRX1*, a class III peroxidase, by *FmNAC104* emerged as the most functionally compelling prediction, given the critical role of peroxidases in the final oxidative polymerization of lignin. Orthogroup analysis showed that the *FmNAC104* orthogroup contained one copy in each examined *Fraxinus* species and no copy in *P. trichocarpa*, whereas the *FmPRX1* orthogroup contained two copies in each examined *Fraxinus* species and one copy in *P. trichocarpa* (Supplementary Dataset S1). These results suggest that the module is better interpreted as a *Fraxinus*-associated and latewood-recruited regulatory pair rather than a strictly *F. mandshurica*-specific family-wide expansion.

To experimentally validate this *in silico* prediction, we performed a comprehensive suite of molecular assays. First, a Yeast one-hybrid (Y1H) assay confirmed that FmNAC104 binds directly to the *FmPRX1* promoter (*pFmPRX1*). Yeast cells co-transformed with *FmNAC104* and *pFmPRX1* grew robustly on selective media containing 3-AT, whereas negative controls (including the non-target promoter *pFmCAD1*) did not (Fig. 7c). Second, a dual-luciferase reporter assay in *Nicotiana benthamiana* leaves demonstrated that FmNAC104 significantly activates the *FmPRX1* promoter *in vivo*. Co-expression of *FmNAC104* resulted in a strong luminescence signal compared to the empty vector control (Fig. 7d, e). Finally, to validate this regulation in the native system, we transiently overexpressed *FmNAC104* in *F. mandshurica* seedlings. qRT-PCR analysis revealed that overexpression of *FmNAC104* triggered a significant (> 3.5-fold) upregulation of the endogenous *FmPRX1* transcript levels (Fig. 7f).

Taken together, these multi-level lines of evidence delineate a latewood-recruited NAC–peroxidase candidate regulatory module. Given the established role of class III peroxidases in the oxidative coupling of monolignols, we propose that recruitment of the *FmNAC104–FmPRX1* module into the latewood developmental program may contribute to latewood-associated lignin polymerization. This module supports a candidate regulatory link between latewood lignification and constitutive structural defense, but direct anatomical validation of cell wall thickening will require further functional studies.

## Discussion

The resilience of long-lived forest trees depends on a sophisticated interplay between structural robustness and effective defense against pathogens. Our high-quality genome assembly of *Fraxinus mandshurica* provides a genomic framework for investigating how structural and immune traits may be coordinated in a long-lived woody species. By integrating genomic, transcriptomic, and functional data, we have identified two temporally associated components of this framework: an expanded and dynamically evolving NLR repertoire with stage-associated transcriptional activity, and a latewood-recruited *FmNAC104–FmPRX1* candidate regulatory module linked to lignification-related secondary metabolism^[104]^. These findings suggest that the exceptional resilience of *F. mandshurica* is not a product of disparate adaptations, but rather a synergistic outcome of a 'fortified' genome architecture that dynamically balances active surveillance with structural integrity.

Our analysis reveals that the expanded NLR repertoire in *F. mandshurica* is predominantly shaped by tandem and proximal duplications^[105]^, which are hotspots for positive selection^[106]^. This observation is consistent with the 'birth-and-death' model of gene family evolution, where localized duplications provide the raw material for new recognition specificities in a co-evolutionary arms race with pathogens^[107]^. Several candidate positively selected NLR pairs appear to have arisen relatively recently, suggesting recurrent diversification of immune receptors in the *F. mandshurica* lineage.

Importantly, NLR copy number and clustering varied among the sampled *Fraxinus* species. This variation suggests that NLR gain/loss and tandem clustering may partly contribute to interspecific differences in immune surveillance capacity; however, receptor copy number alone is unlikely to fully determine realized disease resistance, which also depends on receptor specificity, expression timing, pathogen effector repertoires, and tissue-level structural barriers. Our temporal transcriptomic profiling further suggests a stage-associated deployment pattern: expressed NLRs are enriched during the Transition Stage, a period of rapid xylem expansion and potentially increased tissue vulnerability. This pattern supports the idea that immune surveillance may be transcriptionally reinforced during active xylem development, although direct receptor-effector validation remains an important direction for future work.

As the growing season progresses, the defense strategy shifts from 'active surveillance' to 'structural fortification'. Within this latewood-associated regulatory context, we identified the *FmNAC104–FmPRX1* pair as a candidate NAC–peroxidase module. While NAC-domain proteins are established regulators of secondary cell wall biosynthesis^[108,109]^, our results suggest that *FmNAC104* may participate in the transcriptional regulation of a class III peroxidase during latewood formation^[110]^.

Formal NAC and PRX gene family analysis further clarified the evolutionary status of this module. The NAC repertoire of *F. mandshurica* is larger than those of the examined congeners and *P. trichocarpa* under high-confidence criteria, whereas the PRX repertoire is larger than *P. trichocarpa* and some, but not all, *Fraxinus* congeners. Orthogroup analysis showed that the *FmNAC104* orthogroup is represented by one copy in each examined *Fraxinus* species and absent from *P. trichocarpa*, whereas the *FmPRX1* orthogroup contains two copies in each examined *Fraxinus* species and one copy in *P. trichocarpa*. Thus, this module should be interpreted as a *Fraxinus*-associated and latewood-recruited regulatory pair rather than as a strictly *F. mandshurica*-specific family-wide expansion.

The Y1H, dual-luciferase, and transient overexpression assays support promoter binding, transcriptional activation, and endogenous upregulation of *FmPRX1* by *FmNAC104*. Given the established role of class III peroxidases in monolignol oxidation, these results support a candidate regulatory link between latewood-associated lignin polymerization and structural defense^[111]^. However, direct anatomical validation of cell wall thickening or lignin deposition following stable perturbation of this module remains necessary for future work.

The integrative insight from our study is the temporal integration of these two strategies via shared metabolic flux, which we summarize in our 'Fortified Defense' model (Fig. 8). The phenylpropanoid biosynthesis pathway serves as the critical metabolic hub^[112,113]^. We observed a progressive enrichment of this pathway, culminating in the Latewood Stage. In this model, NLR-associated immune surveillance is transcriptionally active during the Transition Stage, whereas phenylpropanoid and secondary wall-associated programs become more prominent during latewood maturation. The *FmNAC104–FmPRX1* module provides a candidate regulatory link within this late-season program. Rather than representing a fully resolved causal mechanism, the model provides a testable framework for how immune and structural programs may be coordinated during seasonal xylem development in *F. mandshurica*^[114]^.

**Figure 8:**
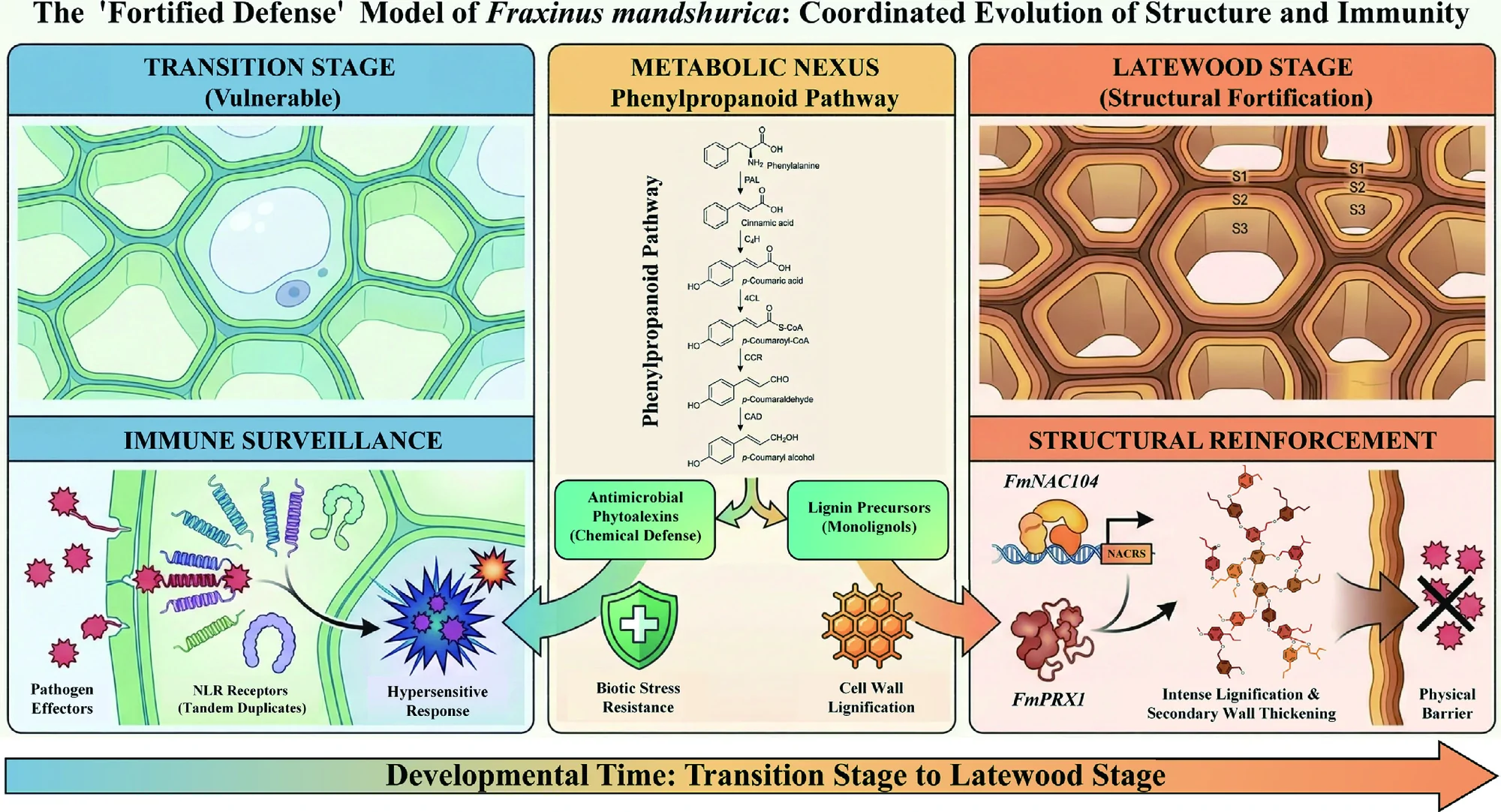
The 'Fortified Defense' working model of *F. mandshurica*. NLR-associated immune surveillance is transcriptionally active during the Transition Stage, whereas phenylpropanoid metabolism and the *FmNAC104–FmPRX1* candidate regulatory module are associated with latewood lignification and structural reinforcement. This model provides a testable framework for the temporal coordination of immune and structural programs during xylem development.

While this study provides a comprehensive genomic and functional portrait of *F. mandshurica*, it also opens several avenues for future research. Although we have validated the core *FmNAC104–FmPRX1* module, the complete regulatory network governing latewood formation is undoubtedly more complex. Future studies combining stable genetic perturbation, lignin quantification, and anatomical measurements of secondary wall thickness will be needed to directly test the phenotypic contribution of this module to latewood fortification^[115]^. Techniques like single-cell RNA-seq^[116]^ or ATAC-seq^[117]^ could fully resolve this hierarchy. Similarly, the curated list of 276 putative effector candidates from *H. fraxineus* predicted in this study (Supplementary Dataset S6) provides a valuable resource for future studies aimed at pairing these effectors with their cognate *F. mandshurica* NLR receptors through high-throughput screening assays^[118]^. Population-level genomic studies across diverse *F. mandshurica* populations will be essential to validate the signals of selection identified in this single reference genome and to trace the demographic history of these adaptive alleles^[119]^.

In conclusion, our work delivers a chromosome-scale genomic resource for *F. mandshurica* and a working framework for understanding how immune-receptor diversification and structural reinforcement may be temporally coordinated in a long-lived forest tree. The identified *FmNAC104–FmPRX1* module and the rapidly evolving NLRs represent prime targets for future tree improvement programs aimed at enhancing both wood quality and disease resistance^[120−122]^.

## Conclusions

We present a chromosome-scale genome assembly of *F. mandshurica*, revealing a candidate framework for temporally coordinated immune and structural programs. Our multi-omics analyses suggest that an expanded and dynamically evolving NLR repertoire is transcriptionally active during the Transition Stage, when developing xylem may be more vulnerable to biotic stress. As the season progresses, the latewood developmental program is associated with phenylpropanoid enrichment and recruitment of the *FmNAC104–FmPRX1* candidate regulatory module, which may contribute to lignin polymerization and structural reinforcement. This spatiotemporal coordination provides a plausible genomic and regulatory basis for the combined wood-quality and disease-resilience traits of the elite germplasm 'M8'. By proposing a 'Fortified Defense' working model, our study offers a new conceptual framework and candidate genomic targets for breeding programs aimed at improving wood quality and disease resilience in *Fraxinus*.

## SUPPLEMENTARY DATA

Supplementary data to this article can be found online.

## Data Availability

The chromosome-scale genome assembly of *Fraxinus mandshurica* clone M8 has been deposited in Genome Warehouse (GWH) under accession number GWHJIVM00000000.1 and is associated with BioProject PRJCA064045 and BioSample SAMC7687003. The genome annotation file has been deposited in Zenodo under doi: 10.5281/zenodo.20140151. The raw sequencing data generated in this study have been deposited in the Genome Sequence Archive (GSA) under BioProject PRJCA064045. Specifically, the genome sequencing-related datasets, including PacBio HiFi, Hi-C, Illumina whole-genome sequencing, and PacBio Iso-Seq reads, are available under accession number CRA043089. The developing xylem transcriptome sequencing data are available under Accession No. CRA043090.
