# Supplementary material for: The Fraxinus mandshurica genome reveals a dual adaptive strategy linking rapidly evolving immune receptors and structural fortification
Source: For Res (Fayettev). 2026 Aug 31;6:e026. doi: 10.48130/forres-0026-0026 (PMC13662928; doi:10.48130/forres-0026-0026)
Supplement: Supplementary file 1 [file forres-2026-6-1-0026-S1.zip › 10.48130_forres-0026-0026-Suppl-FigureS1 (1).pdf]

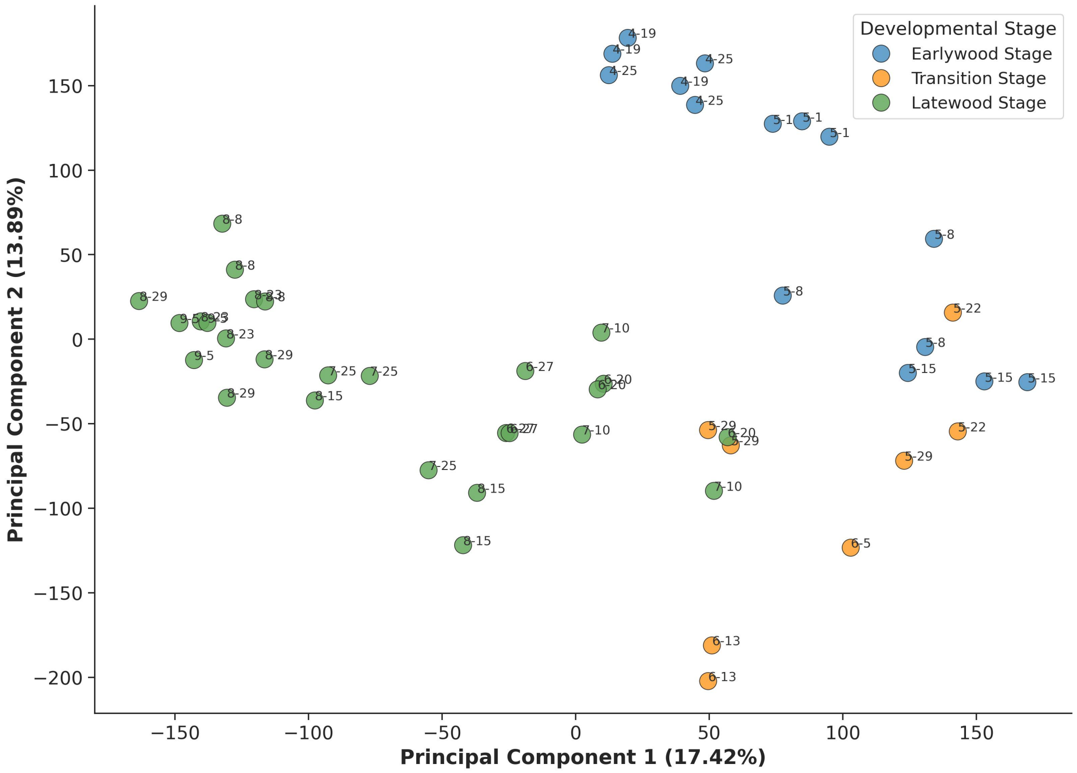

Supplementary Figure S3. Principal component analysis (PCA) of the time-series transcriptomes during *F. mandshurica* xylem development.

The PCA plot displays the global gene expression profiles log_2(TPM+1) of 50 developing xylem samples collected across 18 time points from April 19 to September 5. Biological replicates of each time point are indicated by text labels. Colors represent the three defined developmental phases: Earlywood Stage (blue), Transition Stage (orange), and Latewood Stage (green). The samples exhibit a clear continuous developmental trajectory along the principal components, and the tight clustering of biological replicates demonstrates the high reproducibility and reliability of the RNA-seq data.
