# Supplementary material for: The Fraxinus mandshurica genome reveals a dual adaptive strategy linking rapidly evolving immune receptors and structural fortification
Source: For Res (Fayettev). 2026 Aug 31;6:e026. doi: 10.48130/forres-0026-0026 (PMC13662928; doi:10.48130/forres-0026-0026)
Supplement: Supplementary file 1 [file forres-2026-6-1-0026-S1.zip › 10.48130_forres-0026-0026-Suppl-FigureS1.pdf]

Sample	K-mer number	K-mer Depth	Genome Size(Mb)	Heterozygous Ratio (%)	Repeat (%)
Fraxinus_mandshurica	119996908612	147	786.75	1.07	53.53

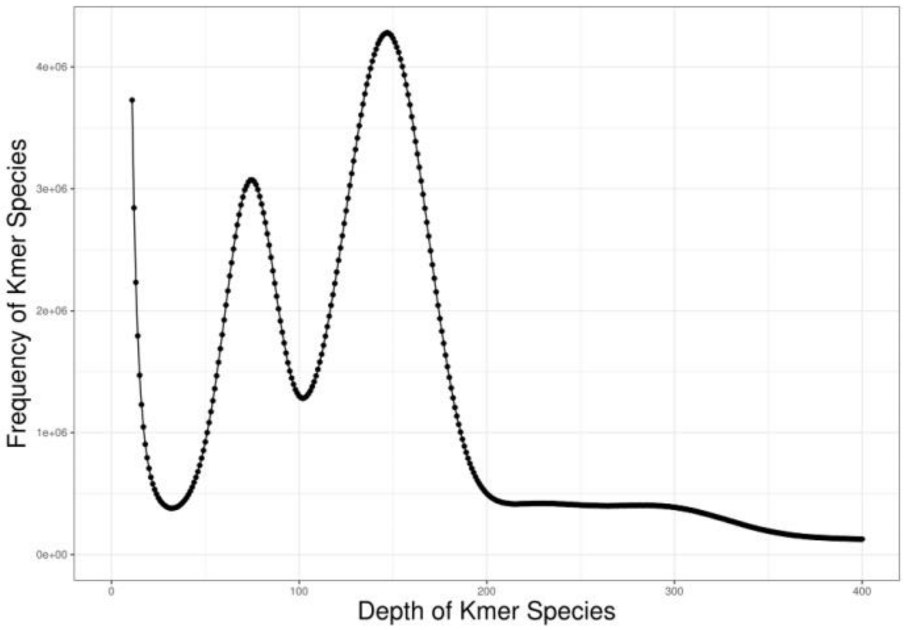

Supplementary Figure S1. K-mer analysis for estimating the genome size and complexity of *Fraxinus mandshurica*.

The distribution of k-mer frequency shows a distinct heterozygous peak and a main homozygous peak. The table above summarizes the estimated genomic characteristics, indicating an estimated genome size of ~786.75 Mb, a heterozygosity rate of 1.07%, and a high repetitive sequence content of 53.53%.
