# Supplementary material for: The Fraxinus mandshurica genome reveals a dual adaptive strategy linking rapidly evolving immune receptors and structural fortification
Source: For Res (Fayettev). 2026 Aug 31;6:e026. doi: 10.48130/forres-0026-0026 (PMC13662928; doi:10.48130/forres-0026-0026)
Supplement: Supplementary file 1 [file forres-2026-6-1-0026-S1.zip › 10.48130_forres-0026-0026-Suppl-FigureS2.pdf]

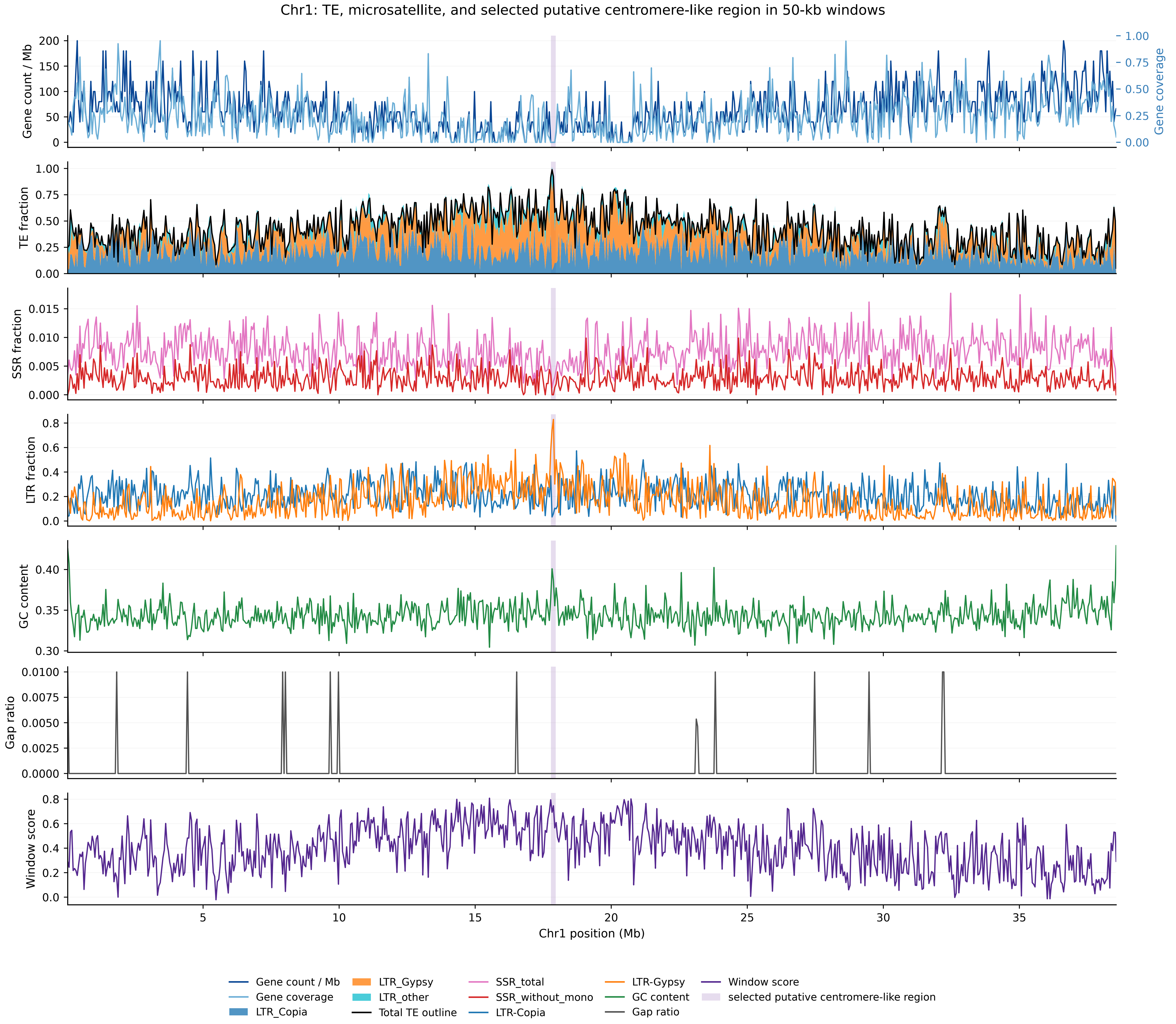

Chr1: TE, microsatellite, and selected putative centromere-like region in 50-kb windows
Gene count / Mb
Gene coverage
TE fraction
SSR fraction
LTR fraction
GC content
Gap ratio
Window score
Chr1 position (Mb)
Gene count / Mb
Gene coverage
LTR_Copia
LTR_Gypsy
LTR_other
Total TE outline
SSR_total
SSR_without_mono
LTR-Copia
LTR-Gypsy
GC content
Gap ratio
Window score
selected putative centromere-like region

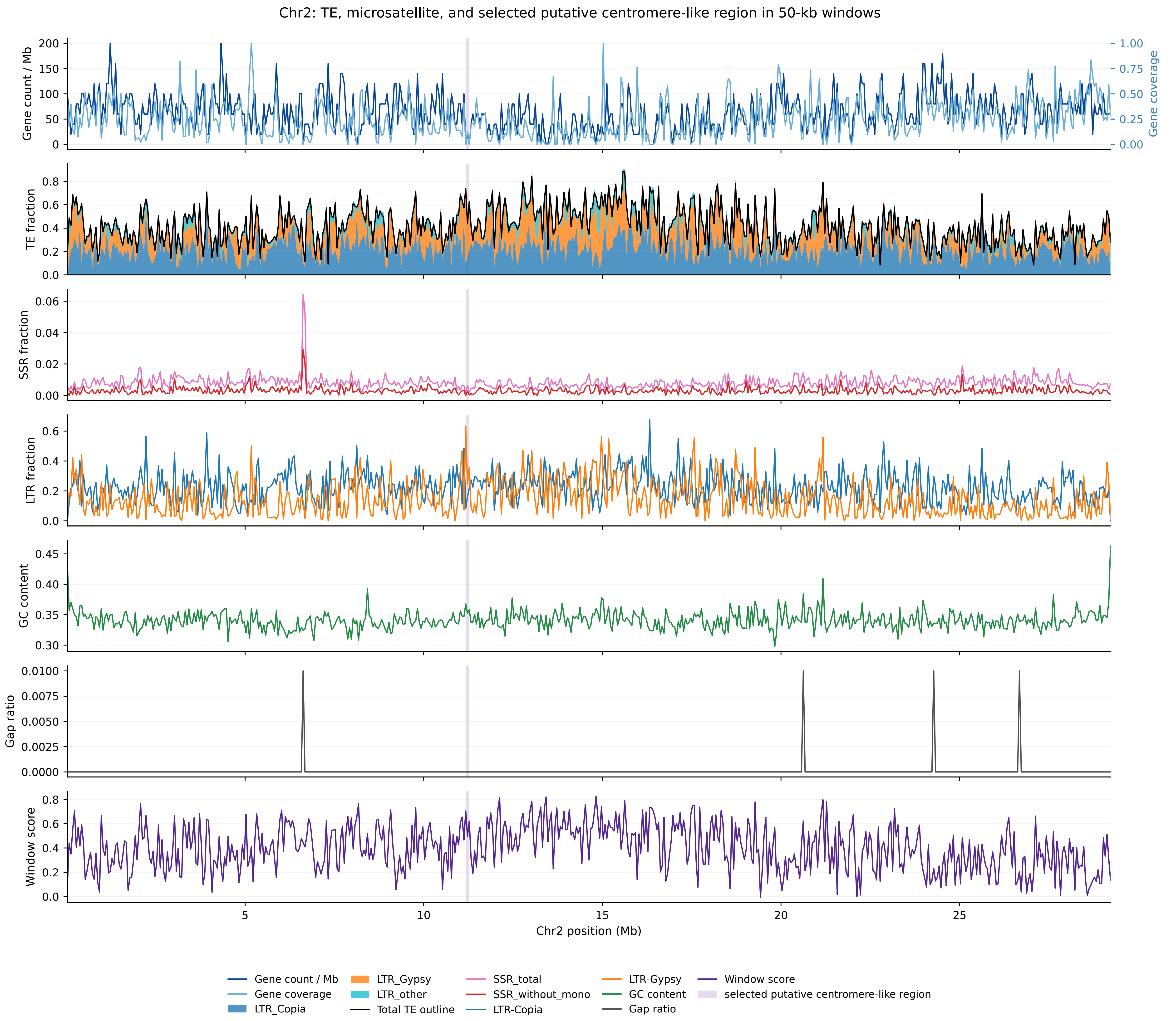

Chr2: TE, microsatellite, and selected putative centromere-like region in 50-kb windows
Gene count / Mb
Gene coverage
TE fraction
SSR fraction
LTR fraction
GC content
Gap ratio
Window score
Chr2 position (Mb)
Gene count / Mb
Gene coverage
LTR_Copia
LTR_Gypsy
LTR_other
Total TE outline
SSR_total
SSR_without_mono
LTR-Copia
LTR-Gypsy
GC content
Gap ratio
Window score
selected putative centromere-like region

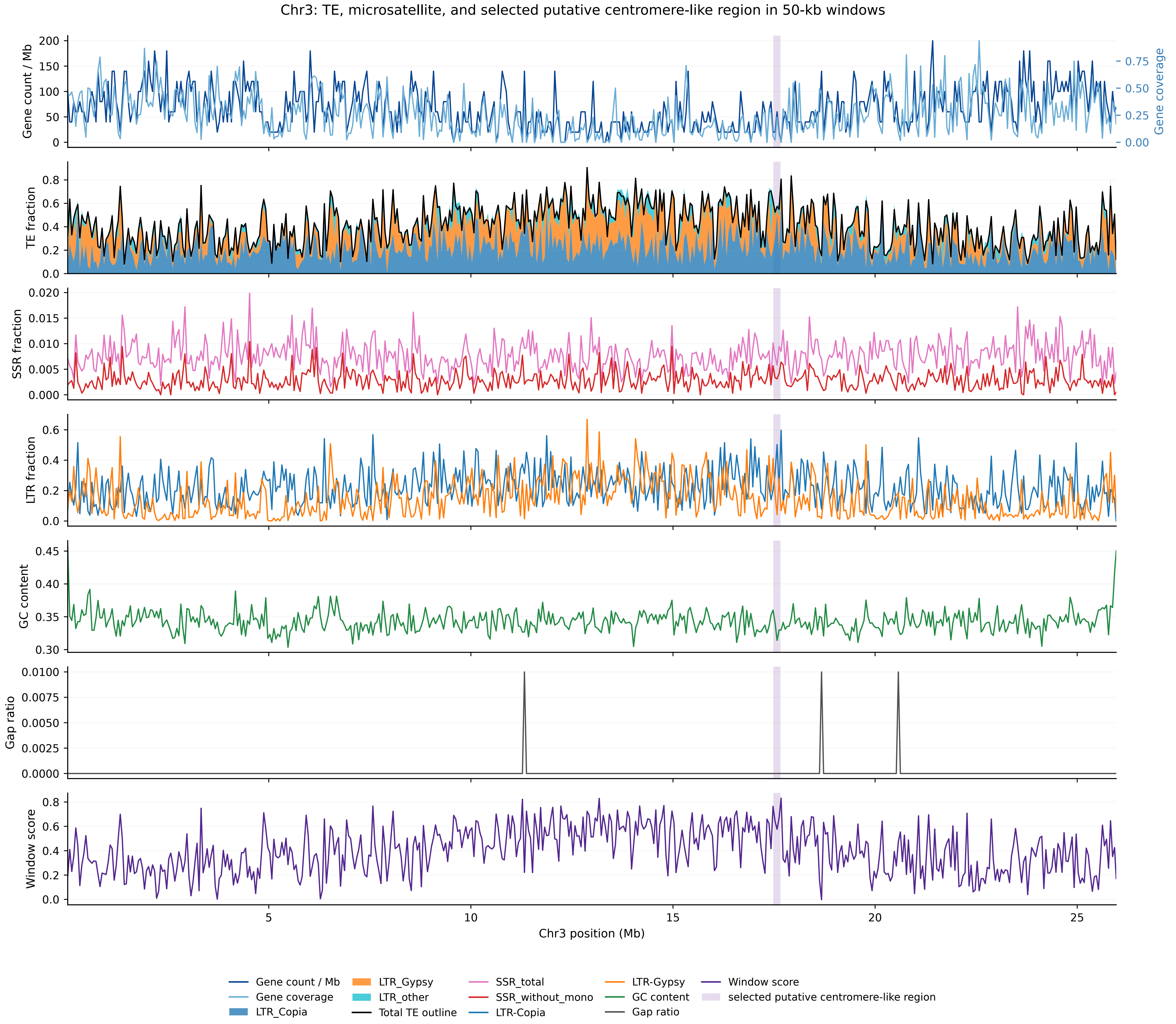

Chr3: TE, microsatellite, and selected putative centromere-like region in 50-kb windows
Gene count / Mb
Gene coverage
TE fraction
SSR fraction
LTR fraction
GC content
Gap ratio
Window score
Chr3 position (Mb)
Gene count / Mb
Gene coverage
LTR_Copia
LTR_Gypsy
LTR_other
Total TE outline
SSR_total
SSR_without_mono
LTR-Copia
LTR-Gypsy
GC content
Gap ratio
Window score
selected putative centromere-like region

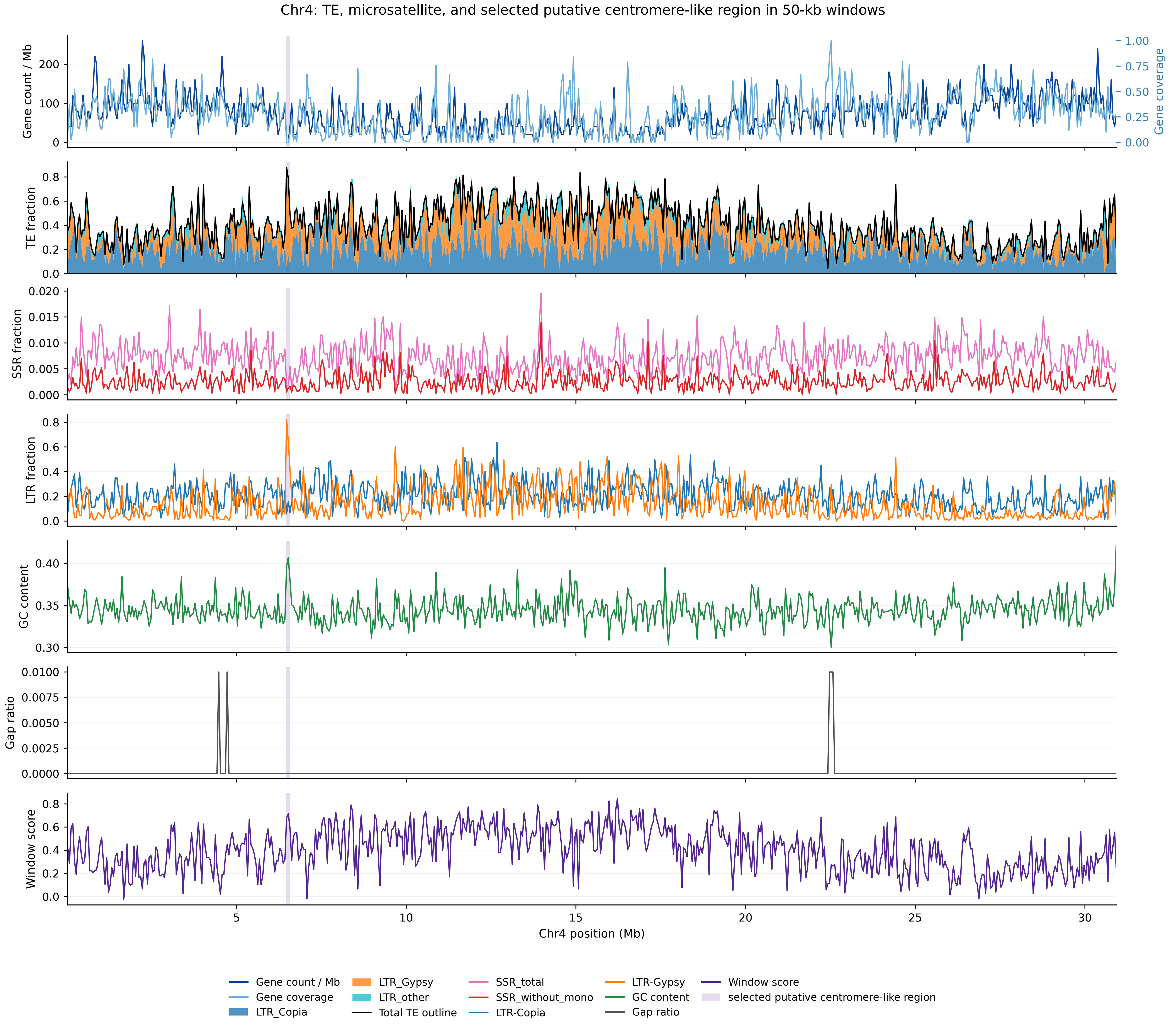

Chr4: TE, microsatellite, and selected putative centromere-like region in 50-kb windows
Gene count / Mb
Gene coverage
TE fraction
SSR fraction
LTR fraction
GC content
Gap ratio
Window score
Chr4 position (Mb)
Gene count / Mb
Gene coverage
LTR_Copia
LTR_Gypsy
LTR_other
Total TE outline
SSR_total
SSR_without_mono
LTR-Copia
LTR-Gypsy
GC content
Gap ratio
Window score
selected putative centromere-like region

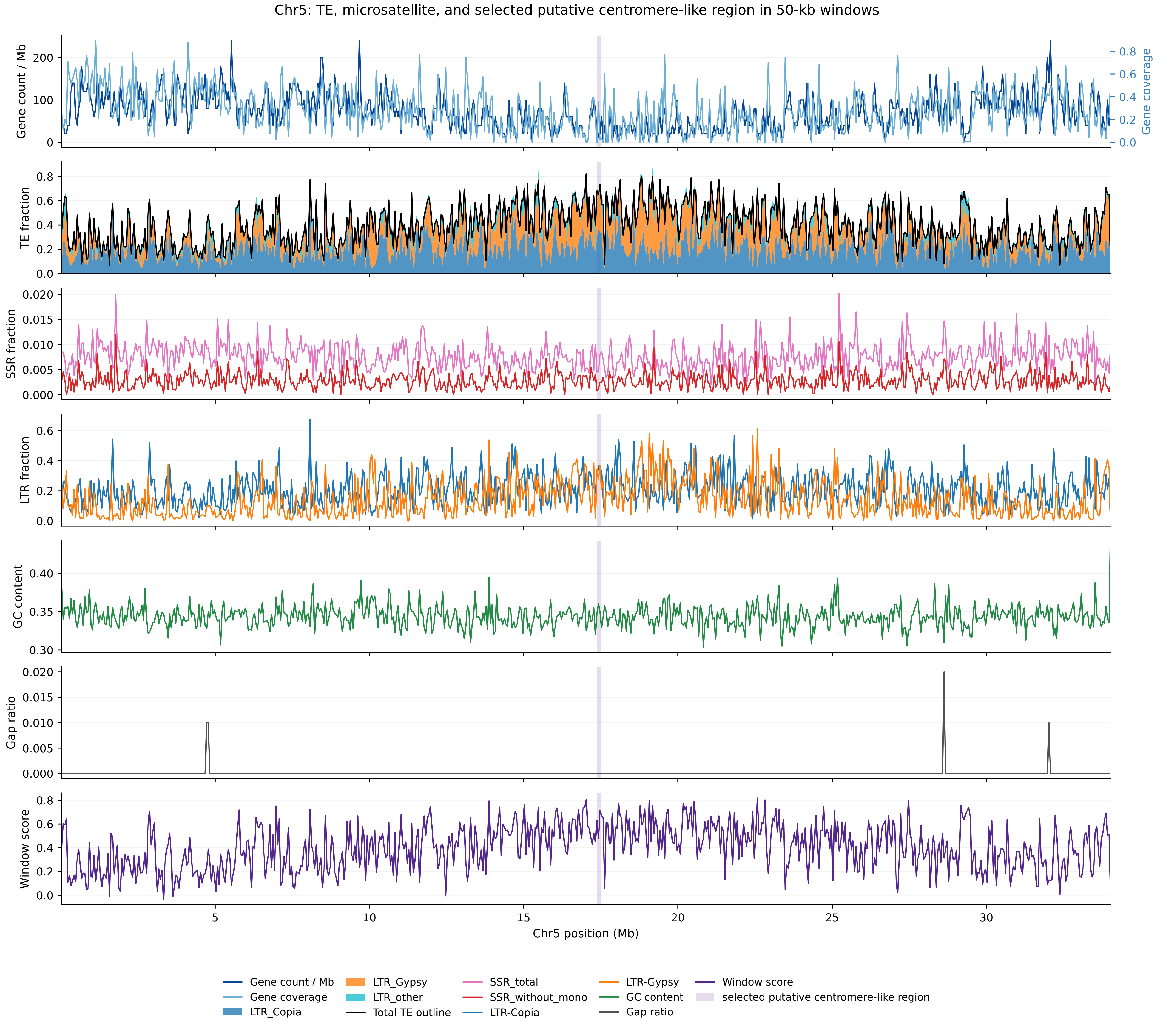

Chr5: TE, microsatellite, and selected putative centromere-like region in 50-kb windows
Gene count / Mb
Gene coverage
TE fraction
SSR fraction
LTR fraction
GC content
Gap ratio
Window score
Chr5 position (Mb)
Gene count / Mb
Gene coverage
LTR_Copia
LTR_Gypsy
LTR_other
Total TE outline
SSR_total
SSR_without_mono
LTR-Copia
LTR-Gypsy
GC content
Gap ratio
Window score
selected putative centromere-like region

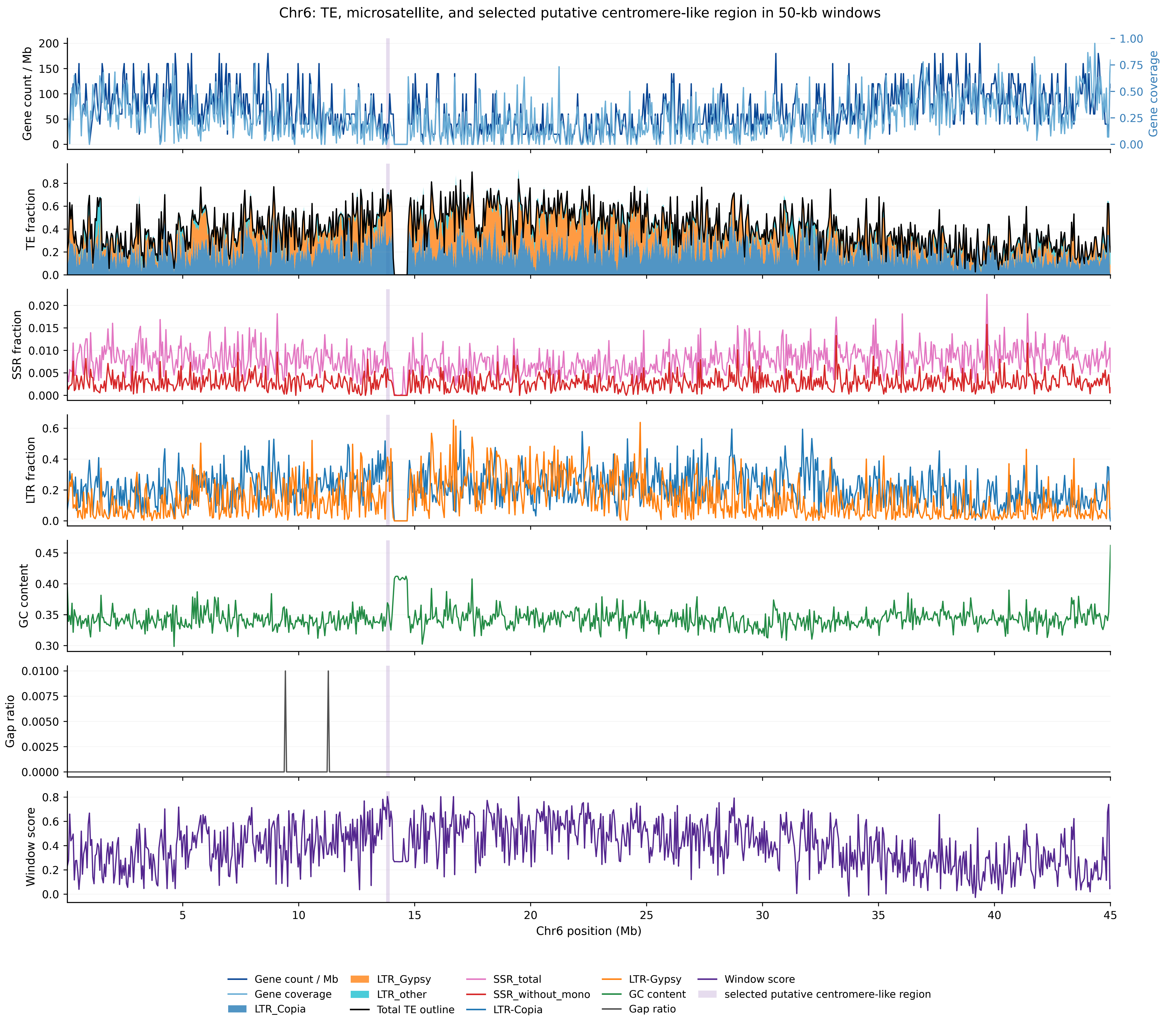

Chr6: TE, microsatellite, and selected putative centromere-like region in 50-kb windows
Gene count / Mb
Gene coverage
TE fraction
SSR fraction
LTR fraction
GC content
Gap ratio
Window score
Chr6 position (Mb)
Gene count / Mb
Gene coverage
LTR_Copia
LTR_Gypsy
LTR_other
Total TE outline
SSR_total
SSR_without_mono
LTR-Copia
LTR-Gypsy
GC content
Gap ratio
Window score
selected putative centromere-like region

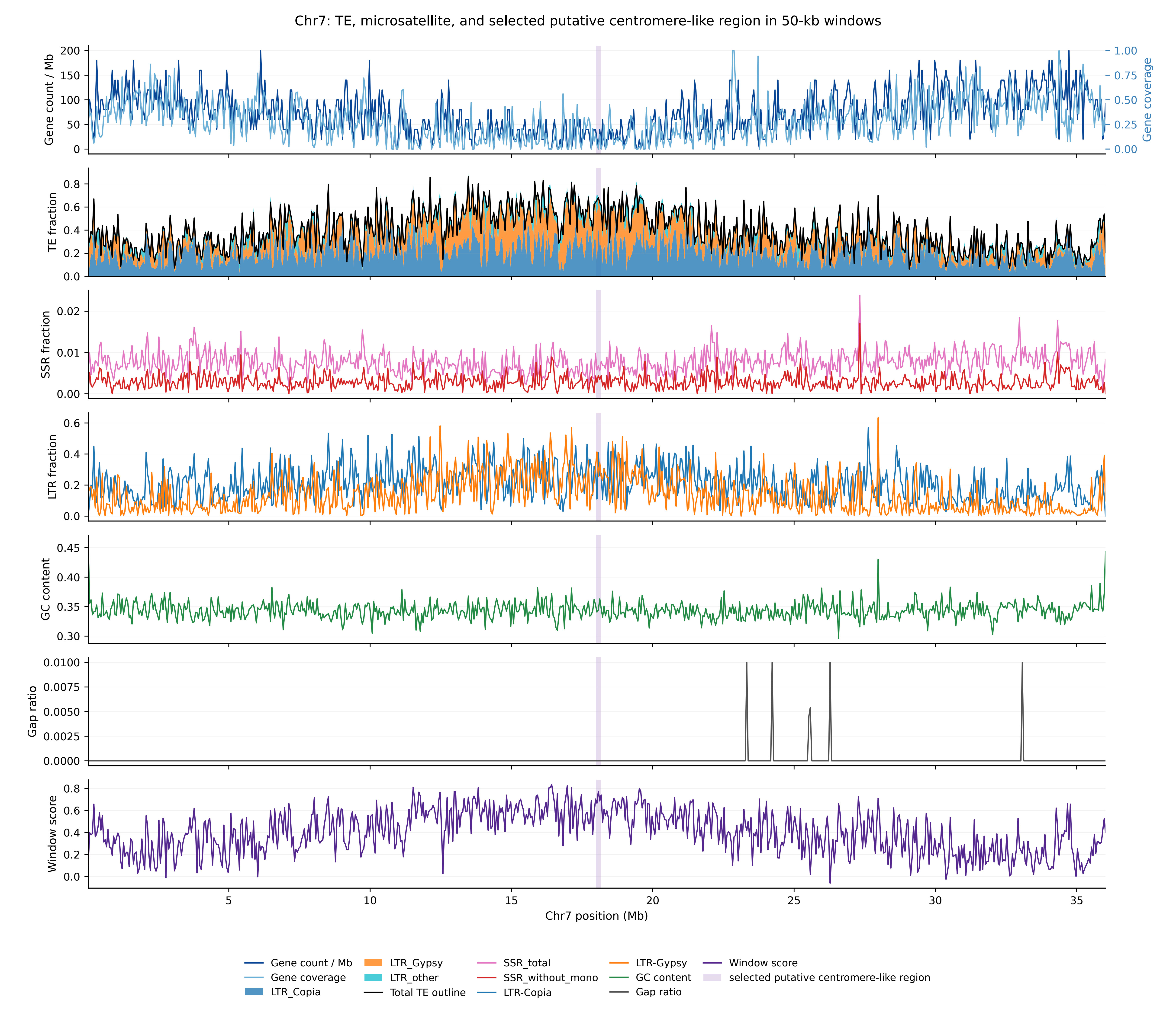

Chr7: TE, microsatellite, and selected putative centromere-like region in 50-kb windows
Gene count / Mb
Gene coverage
TE fraction
SSR fraction
LTR fraction
GC content
Gap ratio
Window score
Chr7 position (Mb)
Gene count / Mb
Gene coverage
LTR_Copia
LTR_Gypsy
LTR_other
Total TE outline
SSR_total
SSR_without_mono
LTR-Copia
LTR-Gypsy
GC content
Gap ratio
Window score
selected putative centromere-like region

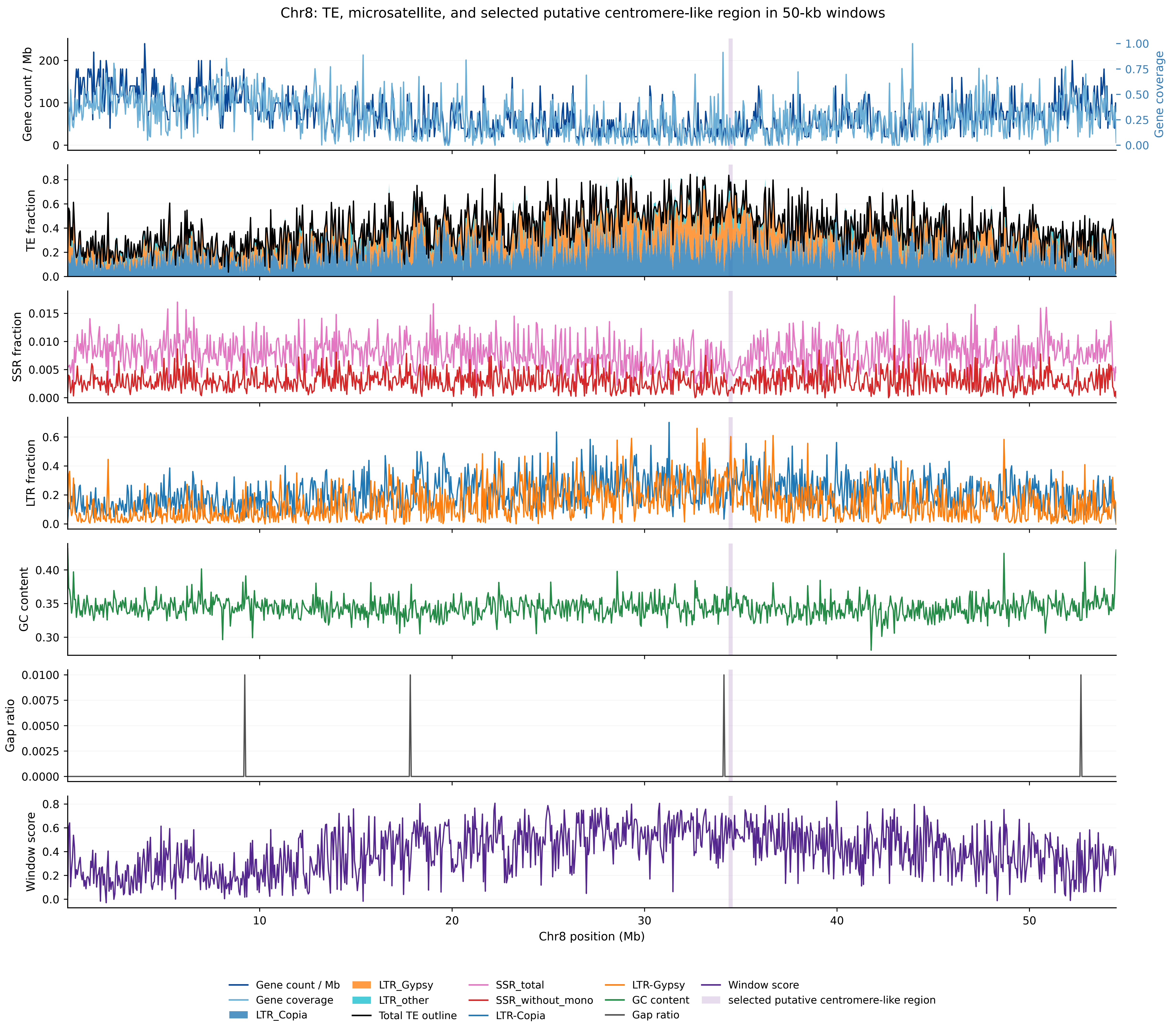
Chr8: TE, microsatellite, and selected putative centromere-like region in 50-kb windows
Gene count / Mb
Gene coverage
TE fraction
SSR fraction
LTR fraction
GC content
Gap ratio
Window score
Chr8 position (Mb)
Gene count / Mb
Gene coverage
LTR_Copia
LTR_Gypsy
LTR_other
Total TE outline
SSR_total
SSR_without_mono
LTR-Copia
LTR-Gypsy
GC content
Gap ratio
Window score
selected putative centromere-like region

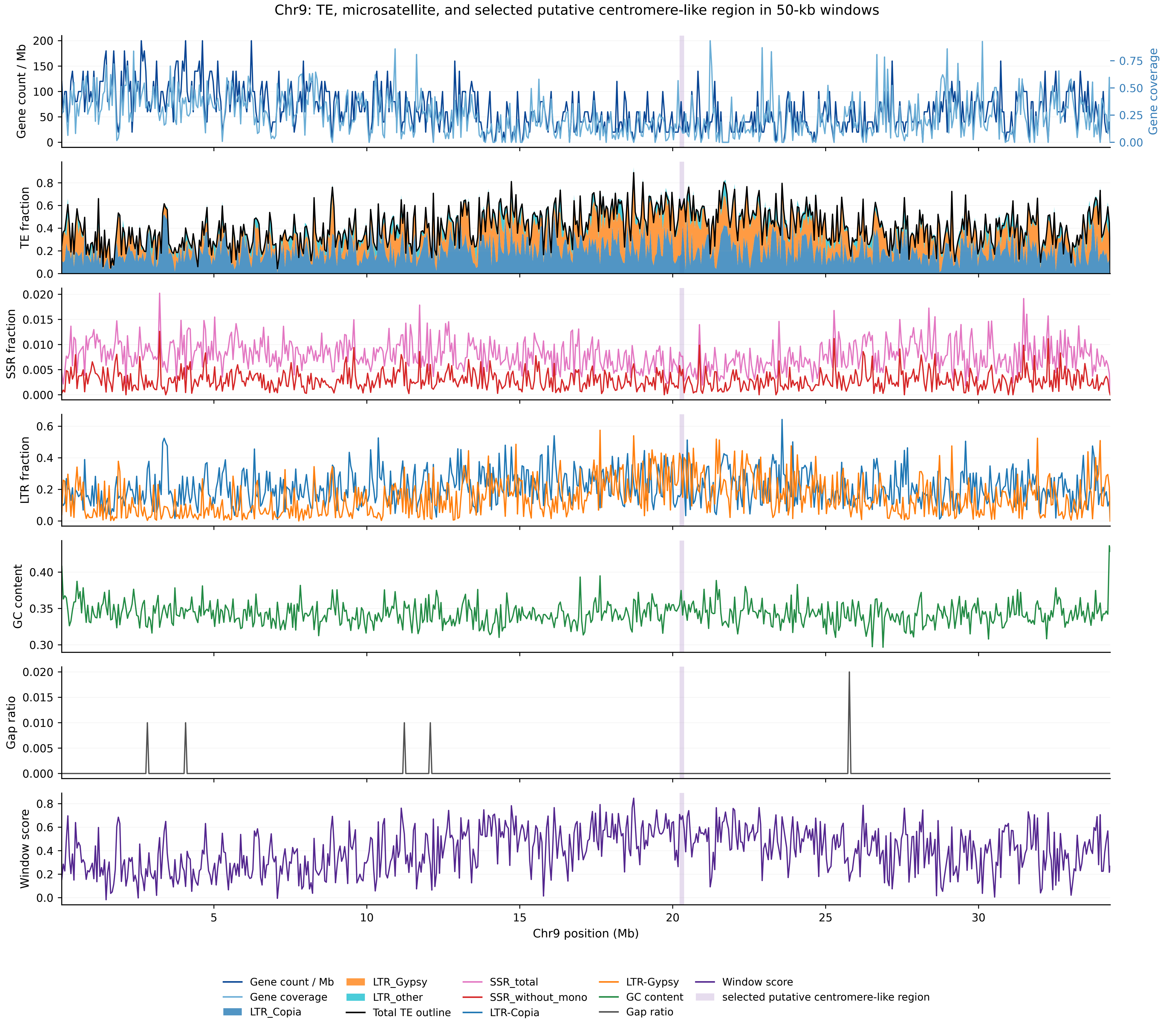

Chr9: TE, microsatellite, and selected putative centromere-like region in 50-kb windows
Gene count / Mb
Gene coverage
TE fraction
SSR fraction
LTR fraction
GC content
Gap ratio
Window score
Chr9 position (Mb)
Gene count / Mb
Gene coverage
LTR_Copia
LTR_Gypsy
LTR_other
Total TE outline
SSR_total
SSR_without_mono
LTR-Copia
LTR-Gypsy
GC content
Gap ratio
Window score
selected putative centromere-like region

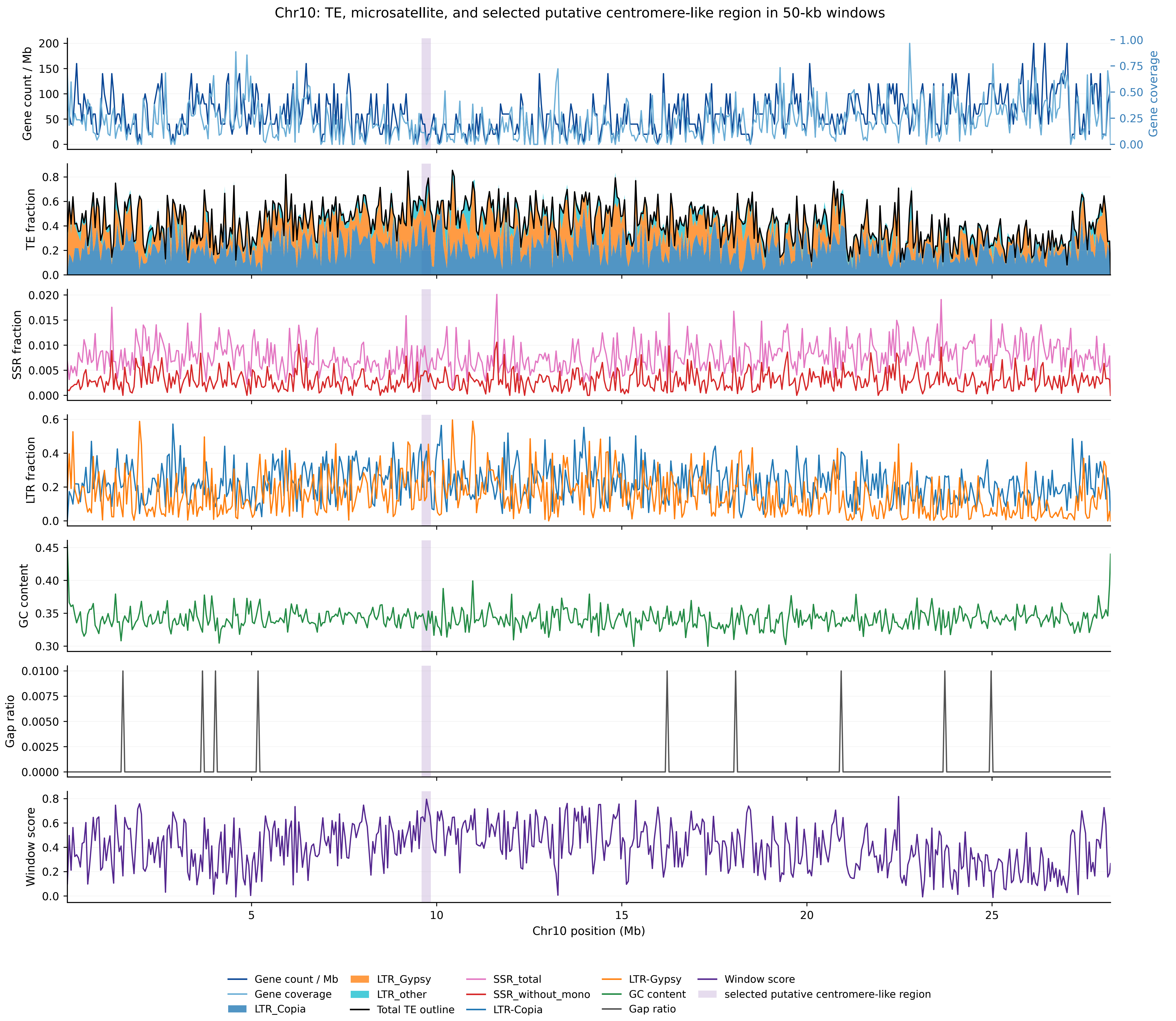
Chr10: TE, microsatellite, and selected putative centromere-like region in 50-kb windows
Gene count / Mb
Gene coverage
TE fraction
SSR fraction
LTR fraction
GC content
Gap ratio
Window score
Chr10 position (Mb)
Gene count / Mb
Gene coverage
LTR_Copia
LTR_Gypsy
LTR_other
Total TE outline
SSR_total
SSR_without_mono
LTR-Copia
LTR-Gypsy
GC content
Gap ratio
Window score
selected putative centromere-like region

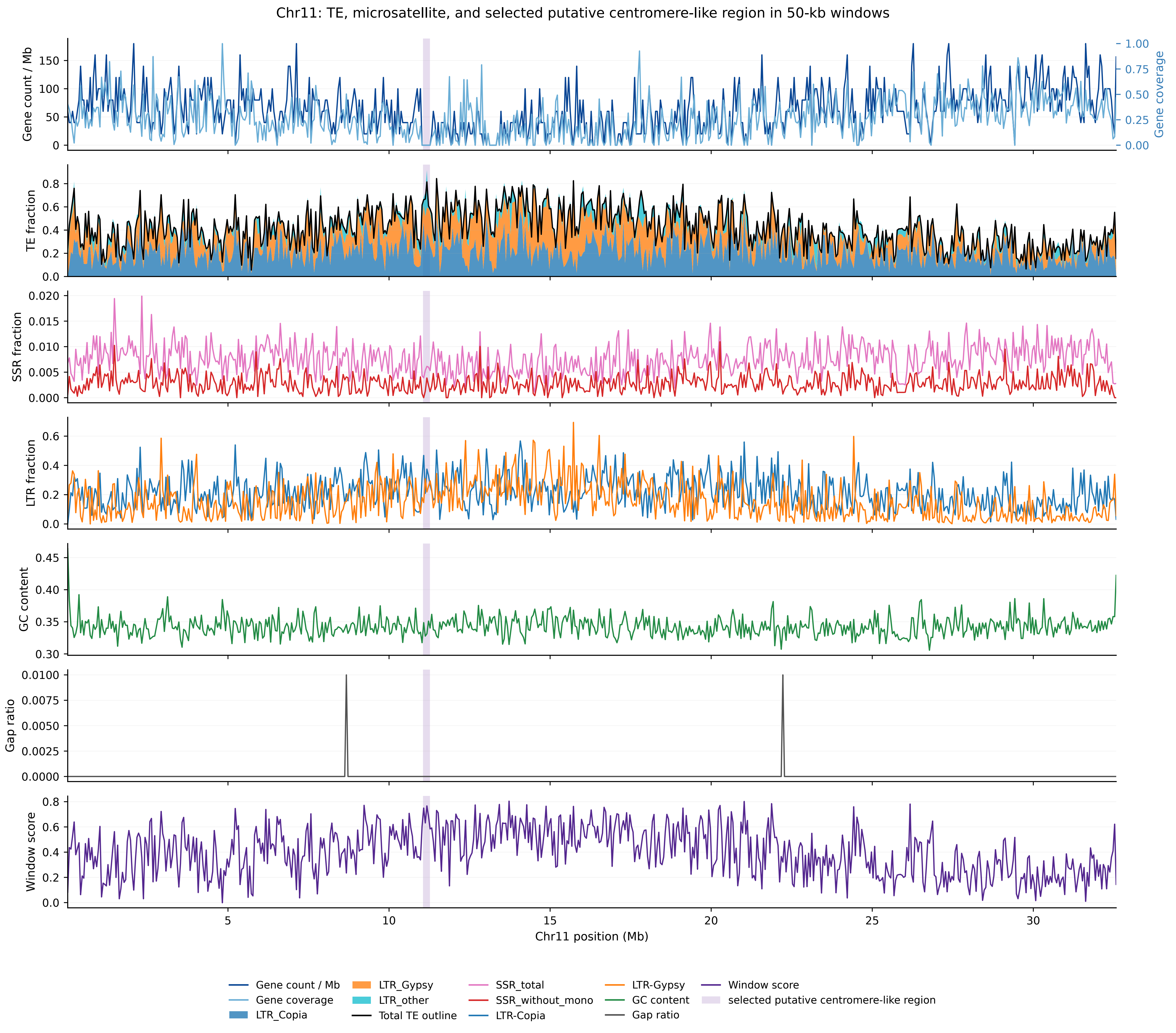

Chr11: TE, microsatellite, and selected putative centromere-like region in 50-kb windows
Gene count / Mb
Gene coverage
TE fraction
SSR fraction
LTR fraction
GC content
Gap ratio
Window score
Chr11 position (Mb)
Gene count / Mb
Gene coverage
LTR_Copia
LTR_Gypsy
LTR_other
Total TE outline
SSR_total
SSR_without_mono
LTR-Copia
LTR-Gypsy
GC content
Gap ratio
Window score
selected putative centromere-like region

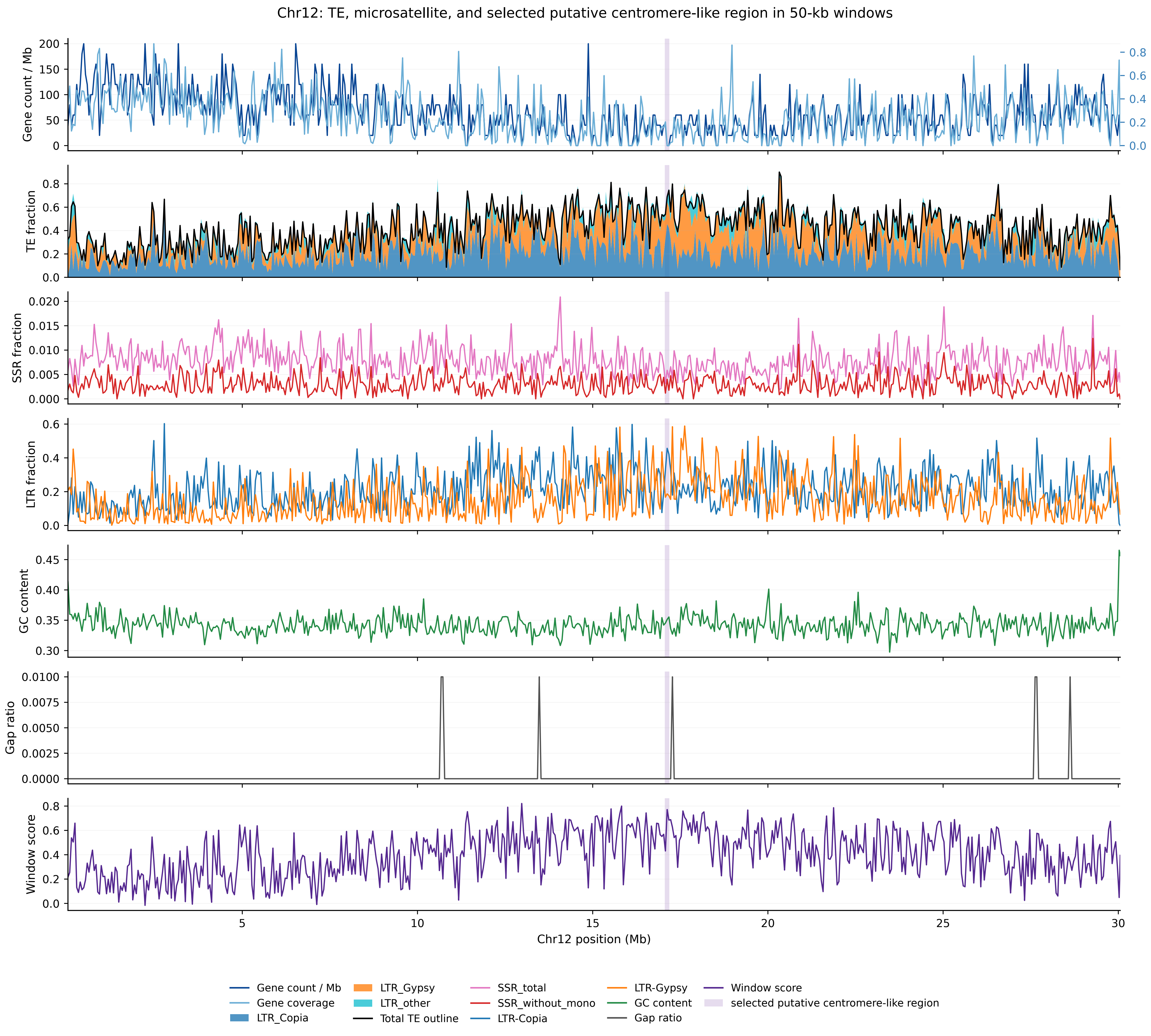

Chr12: TE, microsatellite, and selected putative centromere-like region in 50-kb windows
Gene count / Mb
Gene coverage
TE fraction
SSR fraction
LTR fraction
GC content
Gap ratio
Window score
Chr12 position (Mb)
Gene count / Mb
Gene coverage
LTR_Copia
LTR_Gypsy
LTR_other
Total TE outline
SSR_total
SSR_without_mono
LTR-Copia
LTR-Gypsy
GC content
Gap ratio
Window score
selected putative centromere-like region

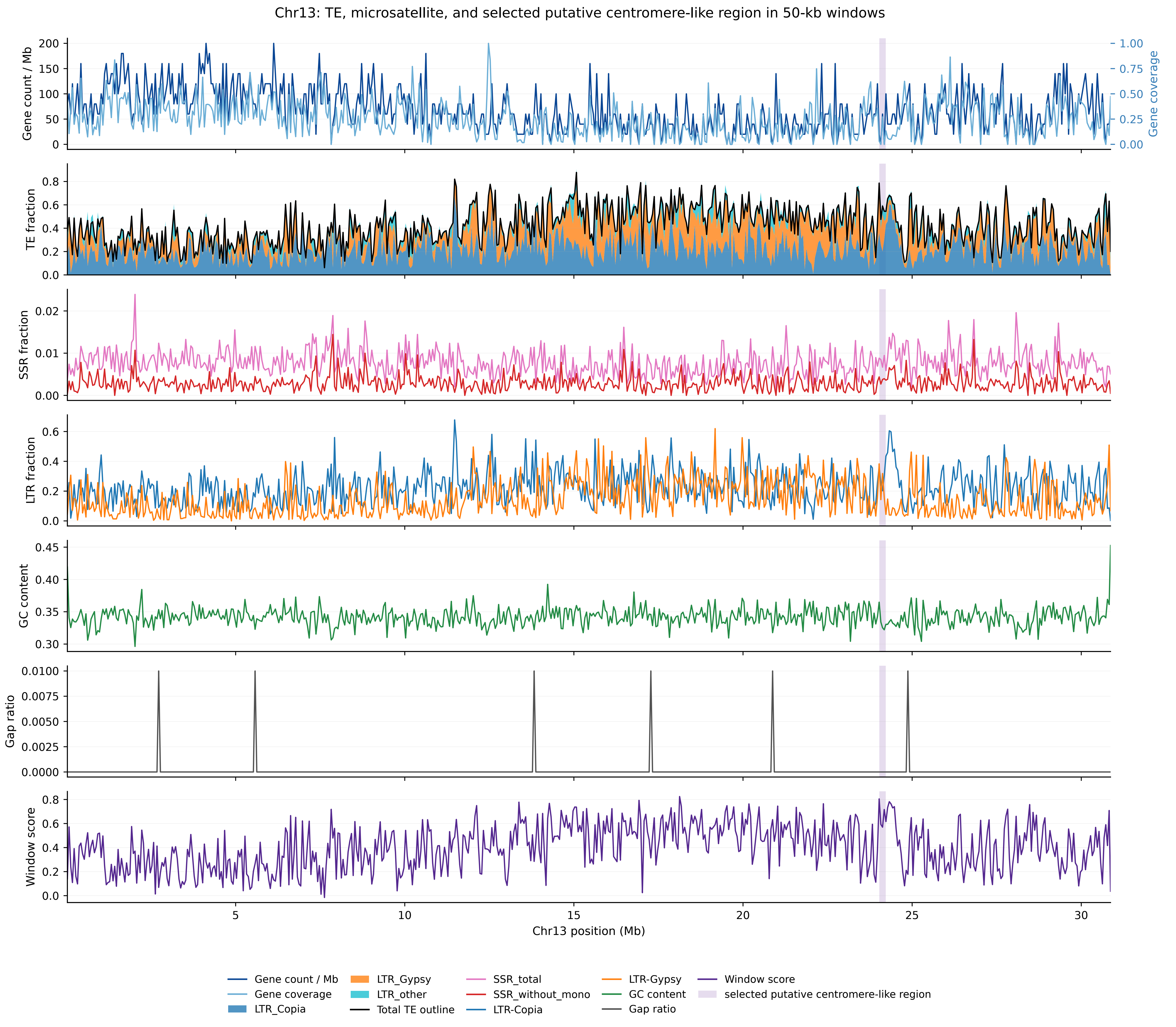

Chr13: TE, microsatellite, and selected putative centromere-like region in 50-kb windows
Gene count / Mb
Gene coverage
TE fraction
SSR fraction
LTR fraction
GC content
Gap ratio
Window score
Chr13 position (Mb)
Gene count / Mb
Gene coverage
LTR_Copia
LTR_Gypsy
LTR_other
Total TE outline
SSR_total
SSR_without_mono
LTR-Copia
LTR-Gypsy
GC content
Gap ratio
Window score
selected putative centromere-like region

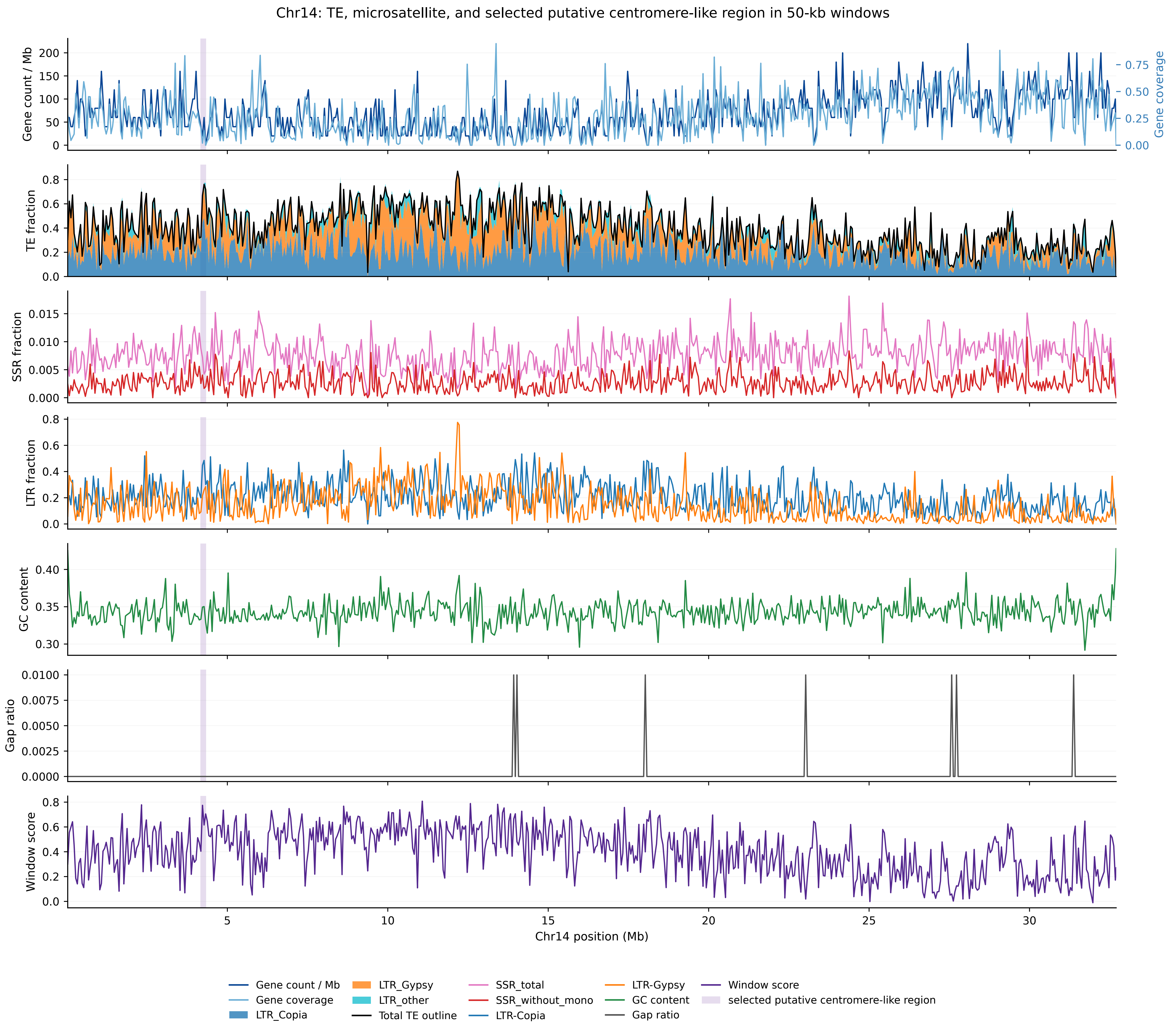
Chr14: TE, microsatellite, and selected putative centromere-like region in 50-kb windows
Gene count / Mb
Gene coverage
TE fraction
SSR fraction
LTR fraction
GC content
Gap ratio
Window score
Chr14 position (Mb)
Gene count / Mb
Gene coverage
LTR_Copia
LTR_Gypsy
LTR_other
Total TE outline
SSR_total
SSR_without_mono
LTR-Copia
LTR-Gypsy
GC content
Gap ratio
Window score
selected putative centromere-like region

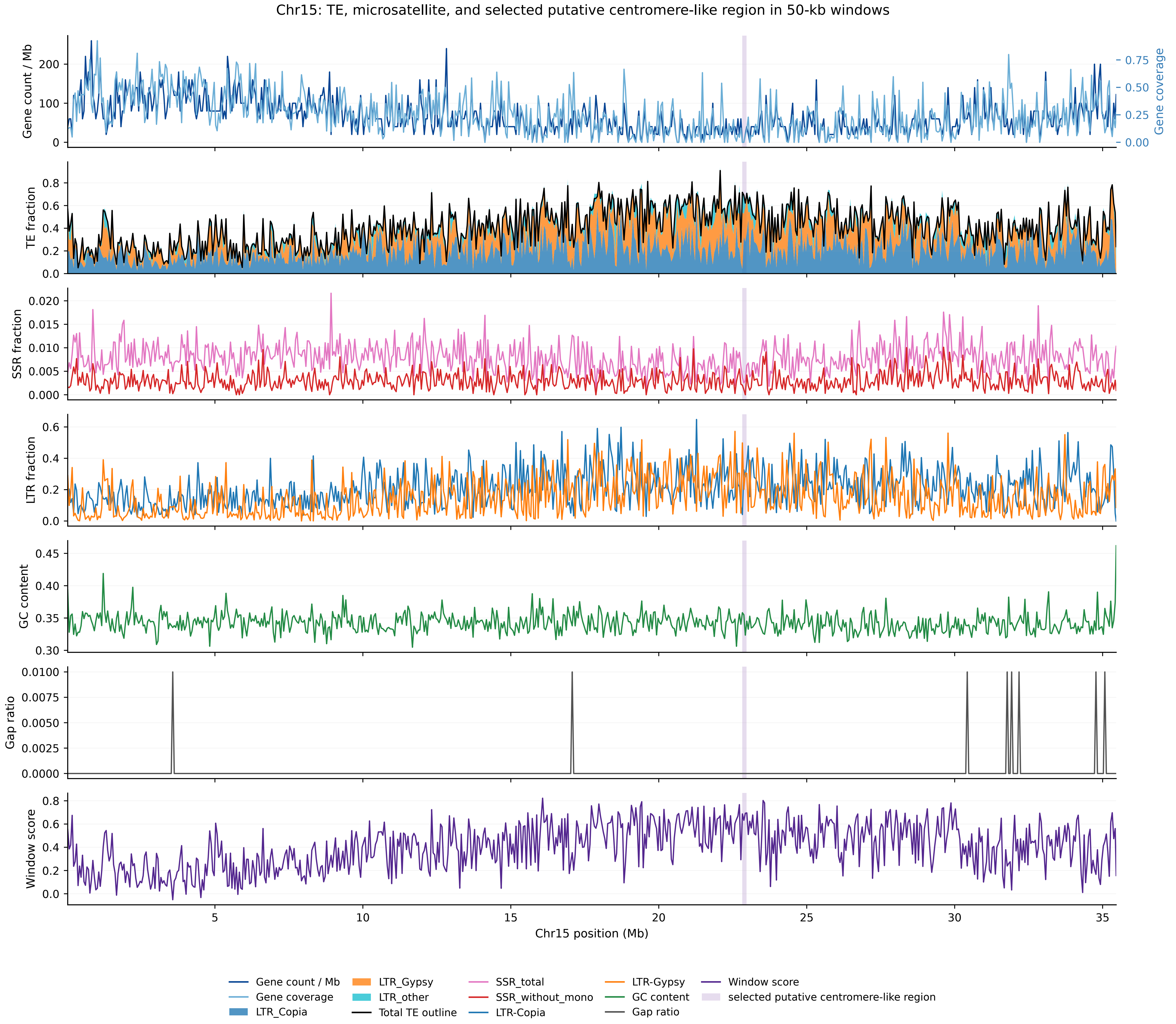

Chr15: TE, microsatellite, and selected putative centromere-like region in 50-kb windows
Gene count / Mb
Gene coverage
TE fraction
SSR fraction
LTR fraction
GC content
Gap ratio
Window score
Chr15 position (Mb)
Gene count / Mb
Gene coverage
LTR_Copia
LTR_Gypsy
LTR_other
Total TE outline
SSR_total
SSR_without_mono
LTR-Copia
LTR-Gypsy
GC content
Gap ratio
Window score
selected putative centromere-like region

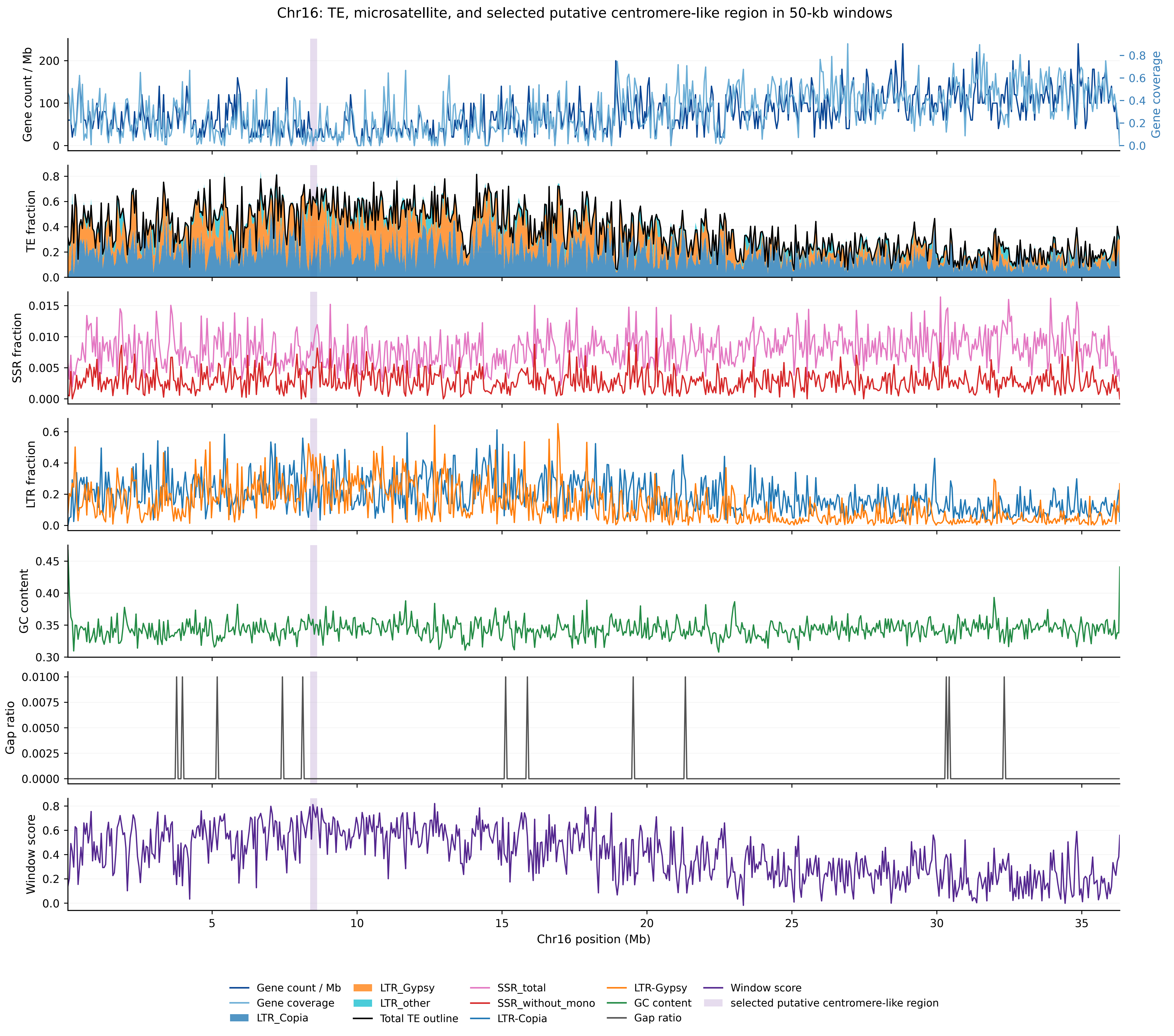

Chr16: TE, microsatellite, and selected putative centromere-like region in 50-kb windows
Gene count / Mb
Gene coverage
TE fraction
SSR fraction
LTR fraction
GC content
Gap ratio
Window score
Chr16 position (Mb)
Gene count / Mb
Gene coverage
LTR_Copia
LTR_Gypsy
LTR_other
Total TE outline
SSR_total
SSR_without_mono
LTR-Copia
LTR-Gypsy
GC content
Gap ratio
Window score
selected putative centromere-like region

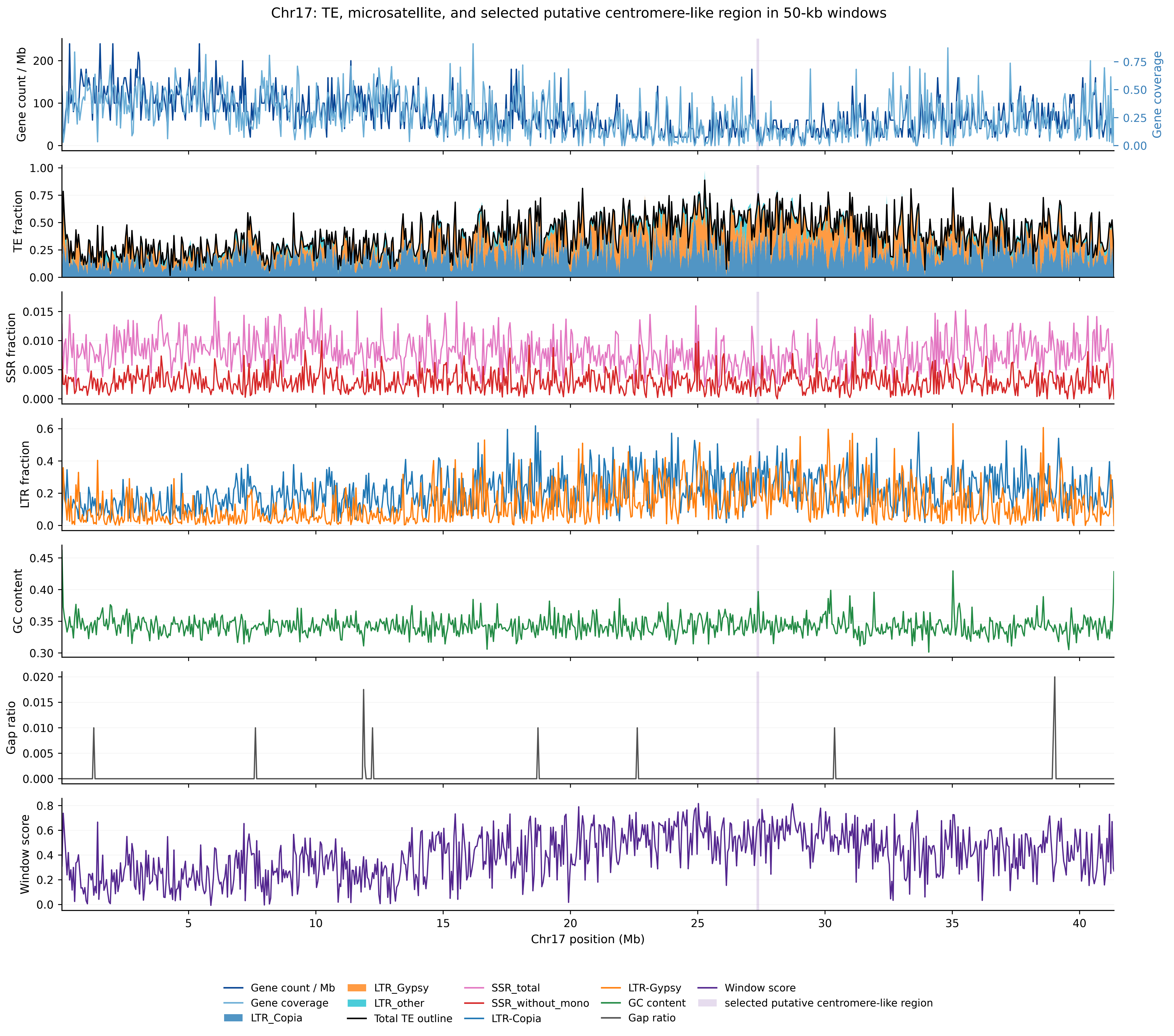

Chr17: TE, microsatellite, and selected putative centromere-like region in 50-kb windows
Gene count / Mb
Gene coverage
TE fraction
SSR fraction
LTR fraction
GC content
Gap ratio
Window score
Chr17 position (Mb)
Gene count / Mb
Gene coverage
LTR_Copia
LTR_Gypsy
LTR_other
Total TE outline
SSR_total
SSR_without_mono
LTR-Copia
LTR-Gypsy
GC content
Gap ratio
Window score
selected putative centromere-like region

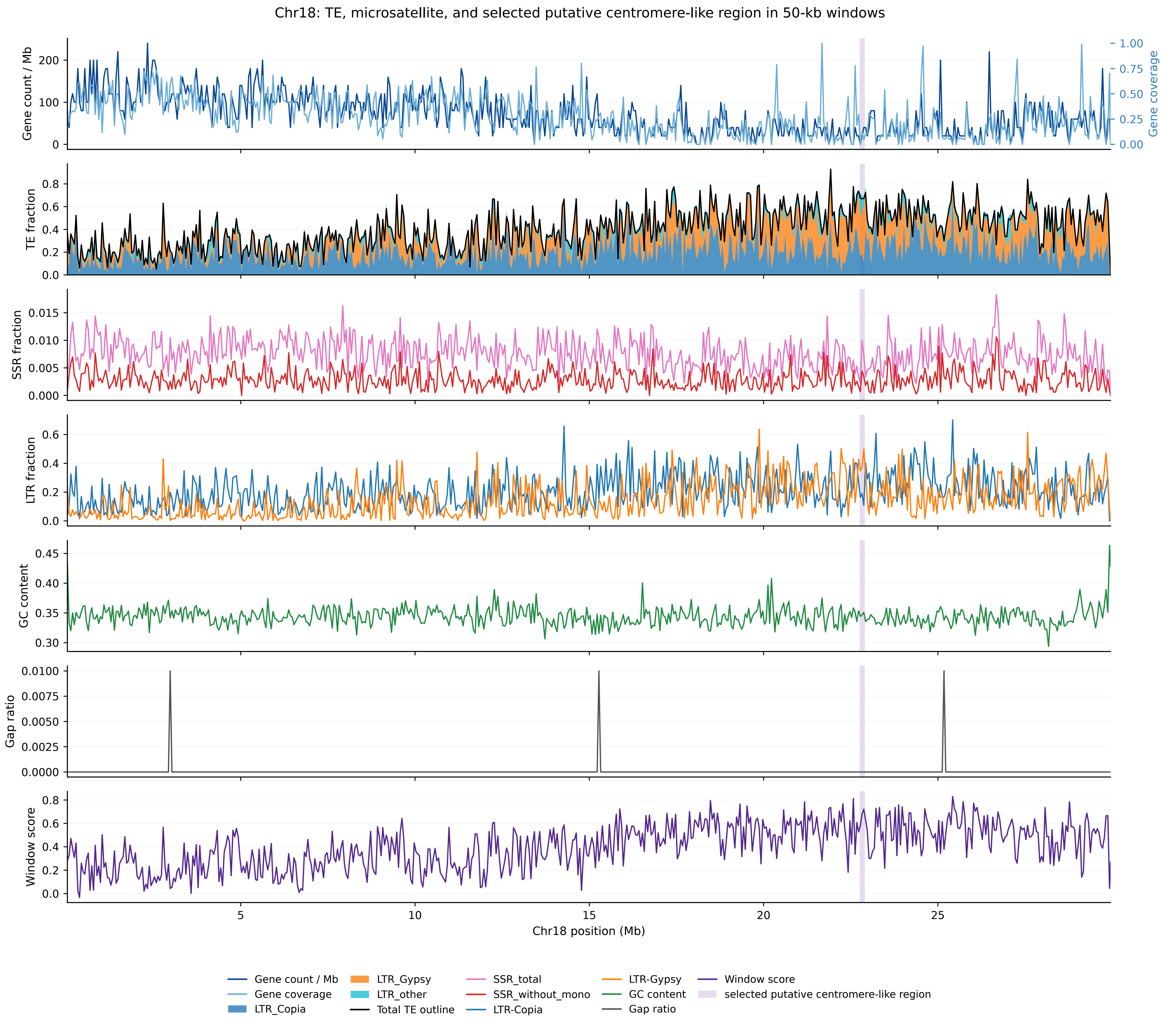

Chr18: TE, microsatellite, and selected putative centromere-like region in 50-kb windows
Gene count / Mb
Gene coverage
TE fraction
SSR fraction
LTR fraction
GC content
Gap ratio
Window score
Chr18 position (Mb)
Gene count / Mb
Gene coverage
LTR_Copia
LTR_Gypsy
LTR_other
Total TE outline
SSR_total
SSR_without_mono
LTR-Copia
LTR-Gypsy
GC content
Gap ratio
Window score
selected putative centromere-like region

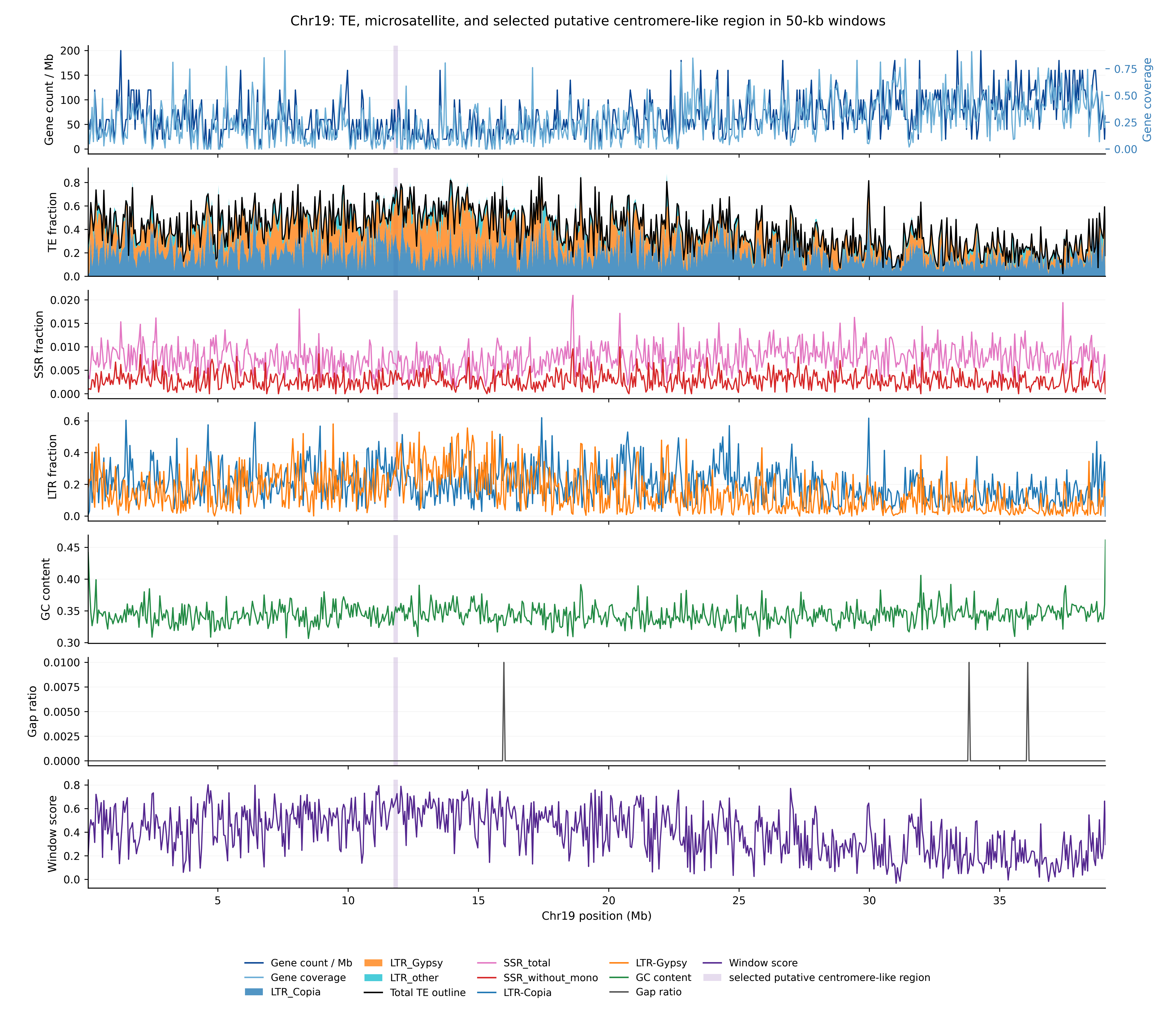

Chr19: TE, microsatellite, and selected putative centromere-like region in 50-kb windows
Gene count / Mb
Gene coverage
TE fraction
SSR fraction
LTR fraction
GC content
Gap ratio
Window score
Chr19 position (Mb)
Gene count / Mb
Gene coverage
LTR_Copia
LTR_Gypsy
LTR_other
Total TE outline
SSR_total
SSR_without_mono
LTR-Copia
LTR-Gypsy
GC content
Gap ratio
Window score
selected putative centromere-like region

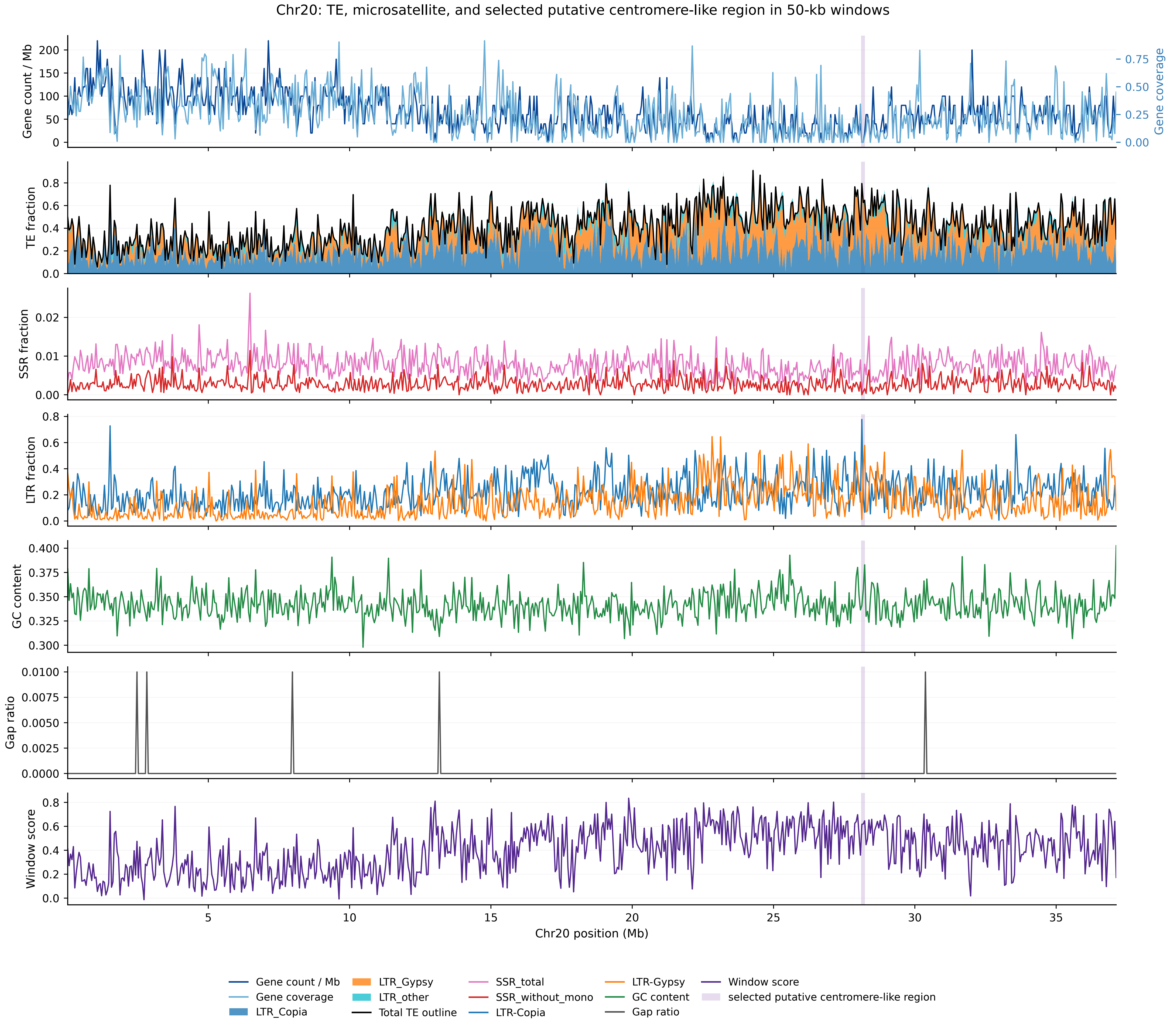
Chr20: TE, microsatellite, and selected putative centromere-like region in 50-kb windows
Gene count / Mb
Gene coverage
TE fraction
SSR fraction
LTR fraction
GC content
Gap ratio
Window score
Chr20 position (Mb)
Gene count / Mb
Gene coverage
LTR_Copia
LTR_Gypsy
LTR_other
Total TE outline
SSR_total
SSR_without_mono
LTR-Copia
LTR-Gypsy
GC content
Gap ratio
Window score
selected putative centromere-like region

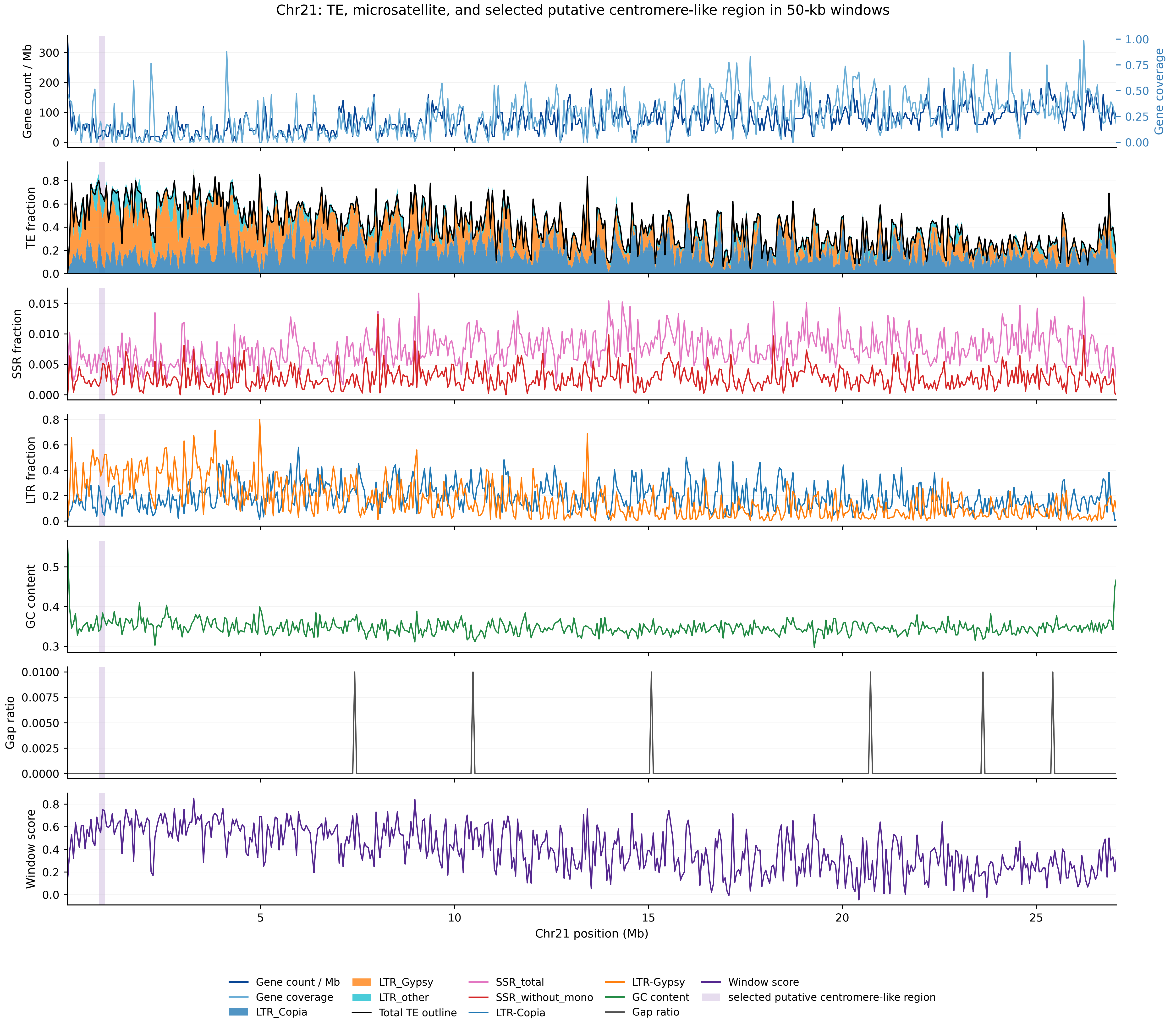

Chr21: TE, microsatellite, and selected putative centromere-like region in 50-kb windows
Gene count / Mb
Gene coverage
TE fraction
SSR fraction
LTR fraction
GC content
Gap ratio
Window score
Chr21 position (Mb)
Gene count / Mb
Gene coverage
LTR_Copia
LTR_Gypsy
LTR_other
Total TE outline
SSR_total
SSR_without_mono
LTR-Copia
LTR-Gypsy
GC content
Gap ratio
Window score
selected putative centromere-like region

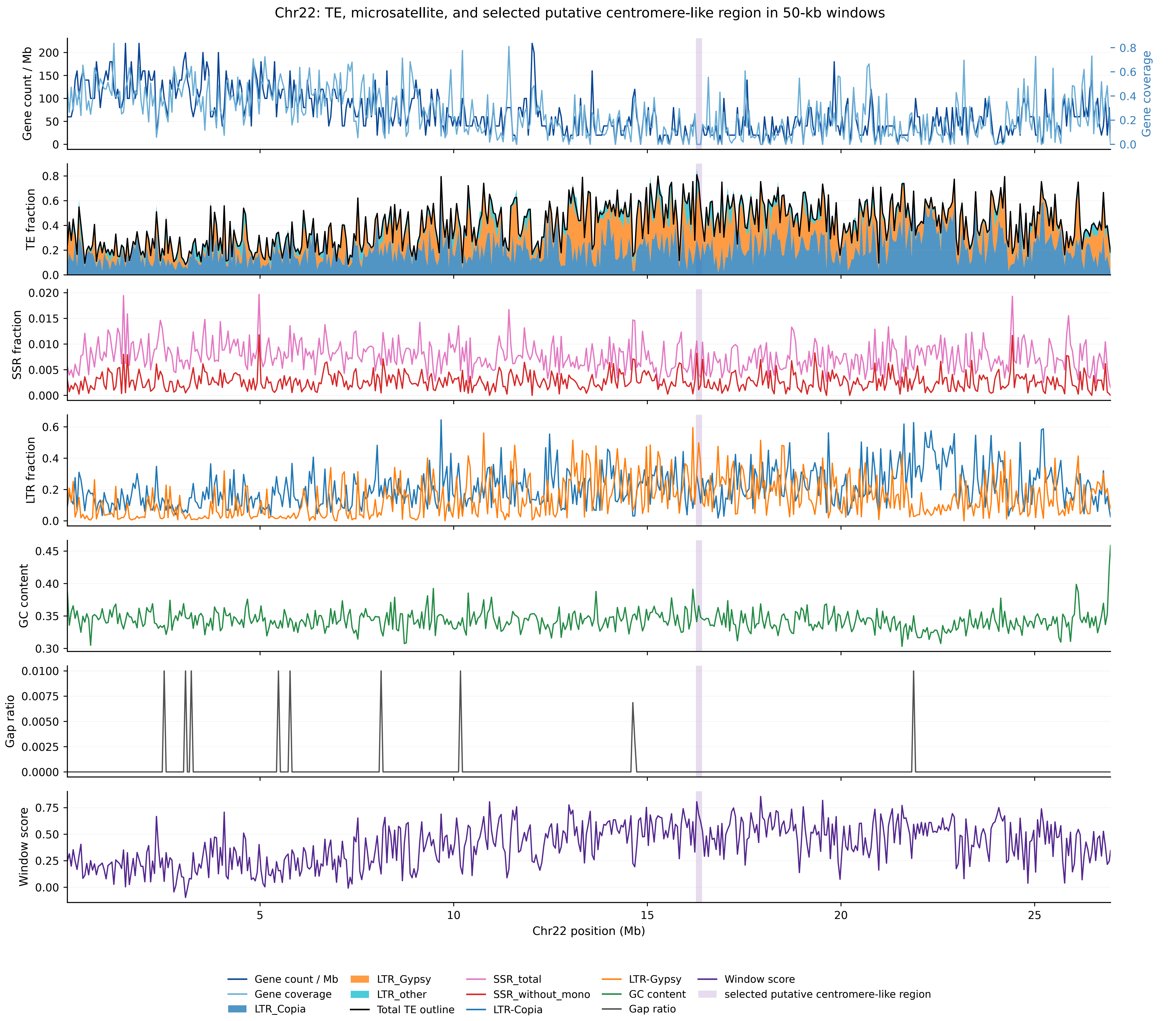

Chr22: TE, microsatellite, and selected putative centromere-like region in 50-kb windows
Gene count / Mb
Gene coverage
TE fraction
SSR fraction
LTR fraction
GC content
Gap ratio
Window score
Chr22 position (Mb)
Gene count / Mb
Gene coverage
LTR_Copia
LTR_Gypsy
LTR_other
Total TE outline
SSR_total
SSR_without_mono
LTR-Copia
LTR-Gypsy
GC content
Gap ratio
Window score
selected putative centromere-like region

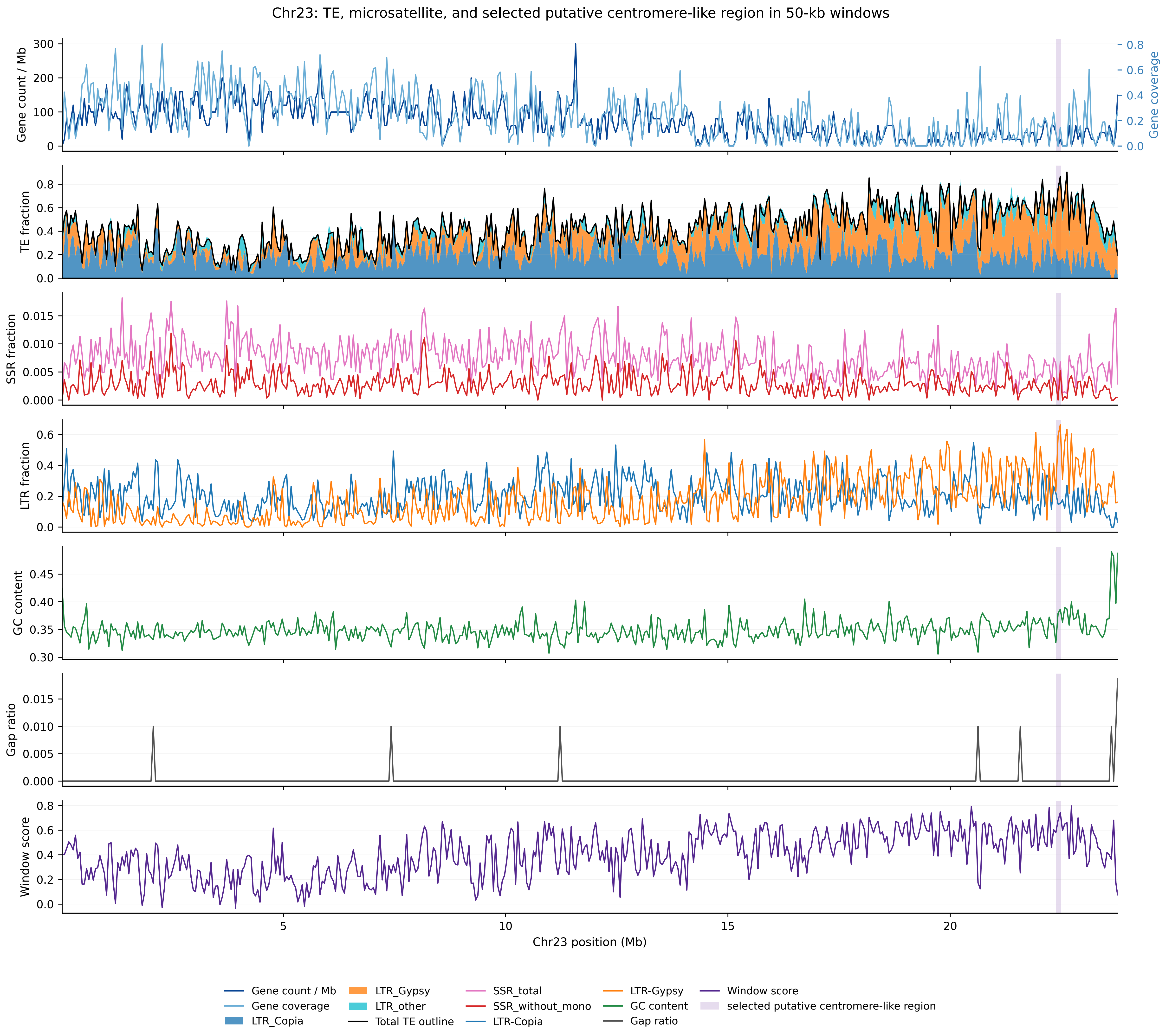

Chr23: TE, microsatellite, and selected putative centromere-like region in 50-kb windows
Gene count / Mb
Gene coverage
TE fraction
SSR fraction
LTR fraction
GC content
Gap ratio
Window score
Chr23 position (Mb)
Gene count / Mb
Gene coverage
LTR_Copia
LTR_Gypsy
LTR_other
Total TE outline
SSR_total
SSR_without_mono
LTR-Copia
LTR-Gypsy
GC content
Gap ratio
Window score
selected putative centromere-like region
